# Fast Myosin Binding Protein‐C Is a Vital Regulator in Young and Aged Fast Skeletal Muscle Homeostasis

**DOI:** 10.1002/jcsm.70106

**Published:** 2025-11-13

**Authors:** Akhil Baby, Kalyani Ananthamohan, Brenna N. McIntyre, Sankar Natesan, Taejeong Song

**Affiliations:** ^1^ Center for Cardiovascular Research, Division of Cardiovascular Health and Disease, Department of Internal Medicine University of Cincinnati Cincinnati OH USA; ^2^ Department of Genetic Engineering, School of Biotechnology Madurai Kamaraj University Madurai India; ^3^ Department of Cellular and Molecular Medicine University of Arizona College of Medicine Tucson Arizona USA

**Keywords:** aging, fast‐twitch muscle fibre, fMyBP‐C, homeostasis, muscle function

## Abstract

**Background:**

Skeletal muscle plays a vital role in voluntary movement and locomotion. Fast‐twitch muscle fibres are characterized by their rapid contraction kinetics, high‐force generation and a distinct gene expression profile compared to slow‐twitch fibres. These fibres have a predominant expression of fast skeletal myosin binding protein‐C (fMyBP‐C). The role of fMyBP‐C in skeletal muscle disease and aging remains poorly understood. To address this, our study employs mouse models with fMyBP‐C ablation to investigate its significance in skeletal muscle physiology.

**Methods:**

Skeletal muscle samples from wild‐type, db/db, MDX and ECC injury model (2–7 months) were analysed to determine the fMyBP‐C levels. Next, male *Mybpc2* knockout (C2^−/−^) mice, both young (3–5 months) and old (22 months), were utilized to investigate the role of fMyBP‐C in aging. The effects of C2^−/−^ and aging on the fibre type, size and number, as well as the overall muscle structure, were evaluated using immunohistochemistry and electron microscopy. In vivo and ex vivo muscle force generation was assessed to determine the functional impact of C2^−/−^ and aging. RNA sequencing was conducted to identify the altered molecular pathways causing the muscle dysfunction in young and old C2^−/−^ mice.

**Results:**

The expression of fMyBP‐C was reduced (0.25‐fold, *p* < 0.05) in the fast‐twitch muscles of db/db mice, with a modest compensatory upregulation of slow skeletal MyBP‐C (sMyBP‐C) (~1.15‐fold, *p* < 0.05). In MDX mice, fMyBP‐C levels remain unchanged, whereas sMyBP‐C levels were upregulated (~1.2‐fold, *p* < 0.01). The fMyBP‐C expression was 75% higher in the male skeletal muscles (*p* < 0.01) compared to females. Studies in young male C2^−/−^ mice revealed a reduction in isometric tetanic force generation by 25% (*p* < 0.01) and relaxation rate by 42% (*p* < 0.001). The C2^−/−^ mice also had 12.8% fewer type IIb fibres (*p* < 0.01), and a 20% reduction in type IIb fibre size (*p* < 0.01). Similarly, aged male C2^−/−^ mice exhibited significant deficits in muscle strength, endurance and survival rate relative to their wild‐type counterparts. The aged male C2^−/−^ mice displayed a reduced size of type IIa, IIx and IIb muscle fibres compared to aged wild‐type mice. RNA sequencing revealed that assembly and trimerization of collagen fibril pathway‐related genes were altered in C2^−/−^ mice.

**Conclusion:**

fMyBP‐C is a critical regulator of muscle function and homeostasis in young male fast‐twitch muscle fibres. Its absence exacerbates the impact of aging on muscle structure and function. These findings suggest that fMyBP‐C could serve as a promising therapeutic target for mitigating muscle wasting associated with aging and disease.

## 1 Introduction

During aging, disruptions in the fast‐twitch muscle homeostasis can significantly impact both muscle function and overall health [1]. Aging is invariably associated with a decline in muscle mass and function, a condition known as sarcopenia. Notably, fast‐twitch muscle fibres are particularly susceptible to age‐related changes, including fibre atrophy and a reduction in contractile function [2, 3]. Similarly, metabolic disorders like diabetes accelerate muscle loss and weakness, possibly mediated by insulin resistance, inflammation, muscle stiffness and neuropathy [4]. In genetic conditions like Duchenne Muscular Dystrophy (DMD), progressive muscle weakness and wasting occur, specifically affecting the structural integrity of fast‐twitch muscle fibres due to a lack of the dystrophin protein [5].

Fast‐twitch muscle fibres, characterized by their rapid contraction kinetics and high‐force generation capacity, are indispensable for executing rapid and forceful body movements [6]. Compared to the slow‐twitch, fast‐twitch fibres have different compositions of key sarcomere elements such as myosin isoform (heavy and light chains) and troponin complex [7]. Myosin binding protein‐C (MyBP‐C) is a sarcomeric regulatory protein located in the C‐zone of the sarcomere's *A*‐band [8]. It interacts with myosin and actin filaments within sarcomeres, modulating cross‐bridge cycling kinetics and regulating force development during muscle contraction [9]. MyBP‐C is also one such protein having a distinct expression profile in slow‐ and fast‐twitch muscles. Among the three MyBP‐C paralogs, fast MyBP‐C (fMyBP‐C) expression is predominant in fast‐twitch muscle fibres, particularly in type IIb, with some presence in type IIx fibres [10].

Mutations in the *MYBPC2* gene, coding for fMyBP‐C, have been implicated in the development of Distal Arthrogryposis myopathy [11]. However, most of the published literature has utilized mouse models to understand the substantial role of fMyBP‐C in enhancing both muscle contractility and integrity [12, 13]. The fine‐tuning of contractile machinery in fast‐twitch fibres is crucial for precisely regulating muscle force and speed. Dysregulation or loss of fMyBP‐C disrupts this control, leading to impaired muscle function, decreased force production and a potential decline in motor performance [12]. However, the precise role of fMyBP‐C specific to fast‐twitch fibres in maintaining homeostasis within aging, injured and diseased muscles, as well as its sex‐specific differences, remains unknown. It is conceivable that the loss of fMyBP‐C and the resultant alterations in muscle structure and function may be instrumental in diminishing muscle force generation and increasing muscle damage, thereby amplifying the morbidity experienced by older individuals. Consequently, understanding the role of fMyBP‐C is essential for elucidating mechanisms underlying muscle contractility and its impact on overall muscle function and regulation.

fMyBP‐C suppresses the myosin transition from OFF to ON state and stabilizes the myosin OFF state [7]. It also maintains the ordered arrangement of the sarcomere by interacting with myosin and titin [14]. Disruptions in fMyBP‐C expression can lead to structural abnormalities, including an increased interfilament lattice spacing, affecting the overall volume of the sarcomere. This compromises muscle integrity, rendering the fibres susceptible to injury and functional impairment [12, 15]. Maintaining muscle integrity is crucial, particularly for older adults who are susceptible to frailty and loss of mobility, which can lead to a poor quality of life [16]. Therefore, the preservation of fMyBP‐C could be crucial for maintaining the structural and functional integrity of the aging fast‐twitch muscles. The current study is aimed at exploring the expression of fMyBP‐C in various disease conditions using diabetic (db/db) and Duchenne muscular dystrophy (MDX) mouse models and assessing the role of fMyBP‐C on muscle homeostasis and age‐related muscle loss using a fMyBP‐C deletion mouse model.

## 2 Materials and Methods

### 2.1 Mouse Models

#### 2.1.1 *Mybpc2* Knockout *(Mybpc2* KO*)* Mouse Model

Two *Mybpc2* KO mouse models were used in this study. The first model (FVBN background) was generated by the targeted replacement of *Mybpc2* exon 2 to 22 with a Neo cassette flanked by two LoxP sites, as previously documented [12]. The second *Mybpc2* KO mouse model (C57BL/6 background) was created by applying the CRISPR/Cas9 technology, targeting exons 6 and 7 of the *Mybpc2* gene. This approach resulted in indel mutations and the deletion of *Mybpc2*, as illustrated in Figure S1. The second mouse model was used for in vivo plantar flexor function tests in young mice and for PKA‐dependent sMyBP‐C phosphorylation experiments. All other data were collected from *Mybpc2* KO mice with an FVBN background (Table S1).

#### 2.1.2 db/db Mice and MDX Mice

The diabetic (db/db, Lepr^db^) and Duchenne muscular dystrophy (MDX, Dmd^mdx^) mice (3–7‐months old) utilized in this study were purchased from Jackson Laboratory (Table S1). For all experimental procedures, mice were anaesthetized using 2.0%–2.5% isoflurane inhalation and euthanized by cervical dislocation before tissue collection. All animal‐related protocols were conducted in strict compliance with the guidelines approved by the Institutional Animal Care and Use Committee at the University of Cincinnati.

### 2.2 Muscle Strength Tests

#### 2.2.1 Grip Strength

Forelimb grip strength was assessed through three trials, during which the mouse was gently pulled against a grip strength meter (1027SM, Columbus Instruments). The maximum recorded value was normalized to the respective body weight and subjected to group‐wise comparisons.

#### 2.2.2 In Vivo Plantar Flexor Force Generation

The isometric tetanic torque of the plantar flexor muscle was assessed in vivo, following the previously established protocol [S1]. With the mouse under anaesthesia using 2.0% inhaled isoflurane, the right knee was immobilized at a 90° angle, and the foot was securely fastened to a footplate connected to a dual‐mode servomotor (Model 300C‐LR, Aurora Scientific). Following the determination of the peak isometric twitch force generation, the isometric tetanic force was recorded at electrical frequencies ranging from 25 to 150 Hz for 350 ms at intervals of 2 min using an Aurora Scientific apparatus (Model 701C).

#### 2.2.3 Ex Vivo EDL Muscle Function

As previously outlined [12], ex vivo EDL muscle contractile properties were assessed in a tissue bath containing oxygenated Krebs–Henseleit buffer (pH 7.4) at 36°C. The proximal tendon was fastened to a secure pin, while the distal tendon was connected to a dual‐mode lever system using silk sutures. Before electrical stimulation, the muscle was allowed to equilibrate in a relaxed state within the bath for 10 min. After determining the peak isometric twitch force (P_t_), we proceeded to measure the peak isometric tetanic force (P_o_) through a series of stimulations ranging from 25 to 200 Hz, with intervals of 2 min. A fatigue test was performed by subjecting the muscle to repeated contractions at 150 Hz, with each contraction separated by 10‐s intervals, for a total amounting to 10 contractions. The physiological cross‐sectional area (CSA) of the muscle was calculated by dividing muscle weight (in grams) by the product of fibre density (1.06 mg/mm^3^) and optimal fibre length (in millimetres). The optimal fibre length was determined by dividing the muscle length by a ratio of 0.44, as described previously [S2]. Specific force (SP_o_) was computed by normalizing P_o_ by CSA (P_o_/CSA) and expressed in units of N/cm^2^. Muscle function data, both in vivo and ex vivo, were digitized using Dynamic Muscle Control (DMC v5.5) software and subsequently analysed employing Dynamic Muscle Analysis (DMA v5.3) software from Aurora Scientific.

### 2.3 Eccentric Contraction (ECC) Induced Muscle Injury

To induce muscle injury, we administered repeated eccentric muscle contractions (ECC, *n* = 100) in the right hindlimb by forcibly inducing dorsiflexion while concurrently generating maximum isometric tetanic torque in the plantar flexors at a frequency of 150 Hz for a duration of 350 ms, with a 5‐s interval between contractions. This was executed at an angle of 14°, with a speed of 0.7° per 10 ms, as previously described [12]. Muscle samples were collected from the gastrocnemius, soleus and plantaris muscles of the injured leg at time points of 0.5‐, 1.0‐, 3.0‐ and 48‐h postinjury in 2 to 3‐month‐old C57BL/6 mice. Contralateral uninjured muscle samples collected at 0.5 h after the injury served as a control.

### 2.4 Sample Collection and Storage

The muscle tissues were dissected, weighed, transferred to labelled cryo‐vials and flash‐frozen in liquid nitrogen for protein and RNA studies. These were stored at −80°C until they were used for experiments. Serum collected from mice post muscle injury was used to analyse the in vivo release profile of sMyBP‐C and fMyBP‐C. Fresh tissue was used to analyse the ex vivo release profile of sMyBP‐C and fMyBP‐C. One day after ECC contraction, the gastrocnemius muscle was dissected and incubated in 800‐μL PBS solution. One hundred microliters of effluent was collected at 0.5, 1.0, 3.0 and 6.0 h after incubation. These samples were stored at −80°C till they were used for ELISA.

### 2.5 Single Muscle Fibre Isolation

EDL muscle was dissected from 2‐ to 3‐month‐old WT and C2^−/−^ mice (*n* = 3). The muscles were incubated in 0.5% collagenase (Roche, #11088807001) for 90 min at 37°C. The digested muscle bundles were transferred to DMEM (Dulbecco's Modified Eagle's Medium) containing 10% horse serum, and the fibres were released by repeated trituration with a glass pipette. Intact fibres were selected and washed twice with DMEM and finally with PBS before proceeding with RNA isolation.

### 2.6 Western Blot Analyses

About 10–20 mg of frozen muscle tissue was dissected and placed in a fresh microtube along with zirconia/silica beads (1.0 mm). The tissue was homogenized in 500 μL of PSB buffer (Biorad, Cat #1632145) with 2 cycles of bead‐beating in a bead mill (BeadBlaster), with a 5‐min incubation on ice between cycles (7 m/s, 60 s). The level of total and phosphorylated proteins was determined following established immunoblotting procedures [12]. Briefly, the protein concentration of the sample lysates was estimated using the BCA assay with albumin standards. About 10–20 μg of each sample lysate was loaded onto 4%–20% precast polyacrylamide gels (BioRad, Mini‐Protean TGX, Cat #4561094) and electrophoresed at 80–100 V for 90 min. Subsequently, the samples were transferred onto nitrocellulose membranes and blocked in 5% nonfat dry milk for 1 h. Primary antibodies, including sMyBP‐C (WH0004604M1 and SAB3501005, Sigma‐Aldrich), fMyBP‐C (SAB2108180, Sigma‐Aldrich), Myh4 (BF‐F3, DSHB), MyoM1 (20360‐1‐AP, Proteintech), Mylpf (A24975, Antibodies.com), Foxo1 (18592‐1‐AP, Proteintech), Ankrd2 (11821‐1‐AP, Proteintech), Cryab (MA5‐27708, Invitrogen) and phospho‐sMyBP‐C (S59/S62 and T190/T191, ProSci Inc., Order # PAS22457 and #PAS22459), were incubated overnight at 4°C. Phospho‐specific sMyBP‐C antibodies targeting Serine 59, Serine 62 and Serine 204 were generously provided by Dr. Kontrogianni‐Konstantopoulos (University of Maryland School of Medicine). Secondary antibodies conjugated with infrared fluorescent dye were incubated for 1 h at room temperature, and protein expression was detected and quantified using the Odyssey CLx system (Li‐Cor). β‐actin (3779, ProSci) or GAPDH (G9545, Sigma) served as a loading control.

### 2.7 ELISA

Standards for sMyBP‐C (BIOMATIK, Cat# EKU06086) and fMyBP‐C ELISA (abbexa, Cat# abx534308) provided in the ELISA kits were resuspended in Standard Diluent buffer to create a 10‐ng/mL standard solution. Serial dilutions of this stock were prepared to yield concentrations of 0.078, 0.156, 0.312, 0.625, 1.25, 5 and 10 ng/mL. Samples were diluted in PBS to 100 μL to standardize volumes, and the dilution factor for final calculations was determined. Both samples and standards were brought to room temperature before use. Duplicates of standards, test samples and control (zero) wells were set on antibody precoated plates, and their positions were noted. Solutions were added to each well bottom without touching the sides. Standards and samples were gently mixed before addition, avoiding foaming. One hundred microliters of diluted standards was added to standard wells, 100 μL of Standard Diluent buffer to control wells and 100 μL of appropriately diluted samples to test sample wells. Plates were mixed, sealed and incubated for 2 h at 37°C. After discarding liquids, 100 μL of Detection Reagent A was added to each well and incubated for 1 h at 37°C. Plates were washed thrice with 1× Wash Buffer, removing any remaining buffer. One hundred microliters of Detection Reagent B was added to each well and incubated for 1 h at 37°C. The wash process was repeated five times, followed by the addition of 90 μL of TMB substrate to each well and incubating at 37°C for 15 min. Fifty microliters of the Stop Solution was added to each well and mixed gently. Thereafter, the absorbance (OD) was measured at 450 nm. Relative OD was calculated as (OD of sample well)—(OD of blank well). A standard curve was plotted using relative OD against the respective concentration of each standard. Sample concentrations were interpolated from the standard curve, and to factor in sample dilution, the concentrations were adjusted by the dilution factor.

### 2.8 PKA‐Dependent sMyBP‐C Phosphorylation

Dissected EDL muscles from WT and C2^−/−^ mice were skinned in a glycerinated relaxing solution containing the following components (in mM): 7 EGTA, 100 BES, 0.017 CaCl2, 5.49 MgCl2, 5 DTT, 15 creatine phosphate, 4.66 ATP, 55.7 K‐propionate, pH 7.0 and 50% (v/v) glycerol. This was performed at 4°C on a gently rotating shaker for 24 h. Muscle tendons were affixed to a wooden stick using silk sutures to secure resting muscle length. The glycerinated relaxing solution was changed thrice during the 24 h, and skinned samples were subsequently stored at −20°C. Four heads of the EDL were then separated and cut into small bundles along their long axis in a relaxing solution. After being washed three times with PBS, the samples were incubated in 200 μL of PKA (0.5 U/μL, Sigma‐Aldrich, P2645) for 1 h at room temperature on a shaker. Skinned muscle fibres were homogenized, and sMyBP‐C phosphorylation was probed using specific antibodies following the aforementioned protocol.

### 2.9 Immunohistochemistry

Immediately after dissection, the EDL and soleus muscles were embedded in O.C.T. compound and flash‐frozen in isopentane cooled with liquid nitrogen. Cross‐sectioned slides, with a thickness of 10 μm, were prepared and stained with H&E or antibodies targeting myosin heavy chains (MHC type I, BA‐D5; MHC type IIA, SC‐71; MHC type IIb, BF‐F3 from DSHB), Pax7 (PA1‐117 from Invitrogen) and laminin (L9393 from Sigma‐Aldrich). Secondary antibodies conjugated with Alexa Fluor (Invitrogen) were used for immunohistology. All images were acquired using a Leica DMi8 and DMI6000 microscopes and analysed with the ImageJ (NIH) software in a blinded manner. The CSA of individual muscle fibres was manually measured using the Freehand Selections tool in ImageJ.

### 2.10 Transmission Electron Microscopy

EDL muscles were fixed through whole‐body perfusion with a solution of 3% paraformaldehyde and 0.1% glutaraldehyde in a 0.1‐M cacodylate buffer for 2 h. Following dissection, the muscles were submerged for 2 h in a solution of 2.5% glutaraldehyde in a 0.1‐M cacodylate buffer with a pH of 7.4 at room temperature. Subsequently, the fixed muscles were sectioned into smaller pieces and subjected to post‐fixation using 1% w/v OsO_4_ dissolved in distilled water. The muscles were then dehydrated gradually using a series of ethanol solutions and finally embedded in Epon resin. Seventy nanometer thickness sections were prepared and initially stained with uranyl acetate followed by lead citrate. Specimens were examined and imaged using a Tecnai G2 Spirit transmission electron microscope.

### 2.11 RNA Isolation and Sequencing Analysis

To compare the effect of *Mybpc2* ablation in the young fast‐twitch muscle, EDL muscle fibres from mice (WT and C2^−/−^) at 2–3 months of age were used. To understand the effect of C2^−/−^ in the context of aging, TA muscle from mice (WT and C2^−/−^), 3–6 and 21–22 months, was used for RNA sequencing. The total RNA was isolated from the snap‐frozen TA muscle or EDL muscle fibres using the RNeasy Mini Kit protocol (Qiagen, Cat #74104) after homogenizing with a bead mill (BeadBlaster). The total RNA was analysed for quantity and quality by spectrometry (Nanodrop ND‐1000, ThermoFisher Scientific) and electrophoresis (Bioanalyzer 2100, Agilent). Only RNA samples with an RIN (RNA Integrity Number) value of 8–10 were used for the sequencing analysis. RNA sequencing was carried out at the BGI Genomics facility. In brief, QC‐passed RNA samples were denatured at suitable temperatures to open their secondary structure, and mRNA was enriched by oligo(dT)‐attached magnetic beads, followed by mRNA fragmentation and cDNA synthesis. The double‐stranded cDNA fragments were subjected to end‐repair, the addition of a single ‘A’ nucleotide to the 3′ ends of the blunt fragments, and ligated to adaptors before amplification. After quality control of the prepared library, the amplified products were denatured to generate single‐stranded PCR products, which were then circularized to replicate using rolling cycle amplification, yielding a DNA nanoball (DNB) containing multiple copies of DNA. Sufficient quality DNBs are then loaded into patterned nanoarrays using the high‐intensity DNA nanochip technique and sequenced through combinatorial Probe‐Anchor Synthesis (cPAS). The raw data containing reads of low quality, connector contamination and excessively high levels of unknown base N were removed before data analysis. The clean reads thus generated were aligned to the reference genome using HISAT (Assembly: GCF_000001635.26_GRCm38.p6). The quantification of raw gene counts was carried out through the utilization of featureCounts (v1.5.2), and subsequent normalization was achieved by applying edgeR's TMM (trimmed mean of M values) method. To identify differentially expressed genes (DEGs), we employed the limma/voom approach. Specifically, genes surpassing the defined moderate threshold criteria (|fold| > 1.5× and (adjusted) *p* < 0.05) were designated as differentially expressed. These DEGs were then employed as queries in EnrichR and ClueGO to retrieve enriched pathways and/or gene ontology terms. Additionally, we performed a GSEA analysis using gene‐set permutation. Additional functional enrichment analysis for DEGs specific and common to young and old C2^−/−^ mice has been carried out using Metascape [S3]. The DEGs and their enrichment data are available in the Supporting Information.

### 2.12 Statistics

The data are reported as the mean ± standard error (SE). Comparisons were conducted using either Student's *t*‐test for experiments with two groups or one‐way ANOVA with Tukey's post hoc test for more than two groups, utilizing GraphPad Prism 7.04 software. Survival curves of WT and C2^−/−^ mice were assessed with the Mantel–Cox test. Statistical significance was defined as a *p*‐value below 0.05.

## 3 Results

### 3.1 Reduced fMyBP‐C Protein Expression in Diseased and Injured Muscles

**FIGURE 1:**
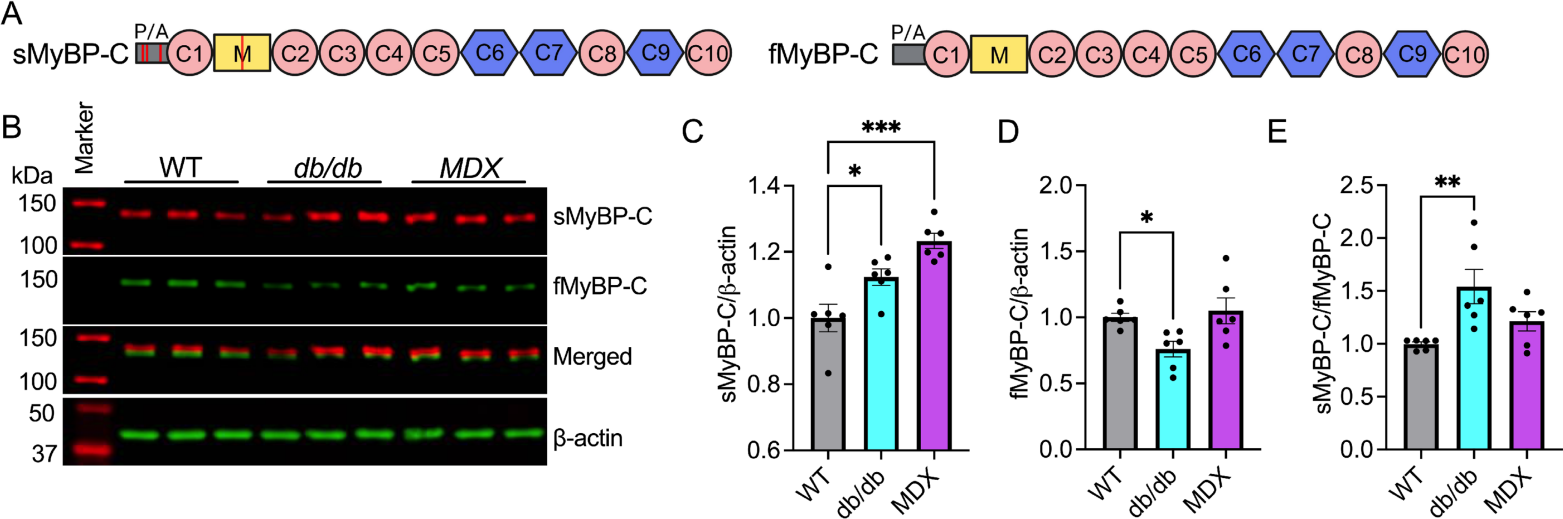
Reduced fMyBP‐C protein level in diseased muscles. (A) sMyBP‐C and fMyBP‐C domain structure (P/A: proline and alanine‐rich region, red circle: immunoglobulin‐like domain, blue hexagon: fibronectin type 3 domain, red line: phosphorylation site). (B) Altered sMyBP‐C and fMyBP‐C protein expressions in wild‐type control (WT) versus diseased (db/db and MDX) tibialis anterior muscles measured by Western blot analysis. Quantitative comparison of the protein expressions between groups (C–E). *n* = 6 mice TA muscles. Mice used were aged 3–7 months. Error bars represent ±SEM and *p < 0.05, **p < 0.01 and ***p < 0.001, db/db vs. WT and MDX vs. WT.

fMyBP‐C expressed in the sarcomeres of fast‐twitch muscle fibres is required for modulating the kinetics of the cross‐bridge cycle and maintaining sarcomere integrity, allowing the precise control of muscle force and speed during rapid high‐force generation [12, 15]. In this study, we first evaluated whether the protein expression of the two skeletal MyBP‐C paralogs (Figure 1A), sMyBP‐C and fMyBP‐C, was altered in diseased and injured muscles. We found a significant reduction of fMyBP‐C in the tibialis anterior (TA) muscle of db/db mice. However, sMyBP‐C levels were significantly elevated in the TA muscle of both db/db and MDX mice models (Figure 1B–D). As a result, the ratio of sMyBP‐C to fMyBP‐C was significantly increased in both diseased muscles compared to the wild‐type (WT) controls (Figure 1E). In the gastrocnemius muscle exposed to eccentric contraction‐induced (ECC) muscle injury, we observed a notable reduction of fMyBP‐C protein level at time points 3‐ and 48‐h post‐injury, whereas sMyBP‐C protein exhibited a more modest decline following the injury (Figure S2C). Notably, fMyBP‐C was released into circulation and detected in serum samples at 1 and 24 h after ECC injury, but we did not see a detectable amount of sMyBP‐C in the same samples (Figure S2D). However, when the muscle at different time points postinjury was incubated in PBS, fMyBP‐C and, to a lesser extent, sMyBP‐C were detected in the 6.0‐h effluent sample (Figure S2E,F). These findings collectively indicate that both absolute and relative fMyBP‐C expression levels were compromised in diseased and injured muscle tissues, and loss of fMyBP‐C may contribute to the functional deficits in these muscles.

### 3.2 Distinct Slow and Fast MyBP‐C Expression Profiles in Male and Female Muscles

**FIGURE 2:**
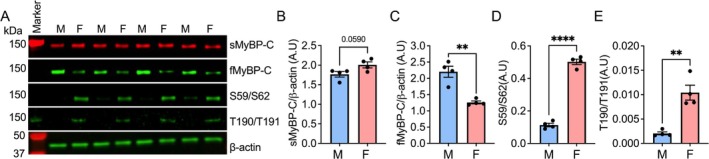
Differential fMyBP‐C expression in male and female skeletal muscle. Represent Western blot images (A), quantifications of sMyBP‐C (B), fMyBP‐C (C), and phosphorylation of sMyBP‐C at Serine 59/62 (D) and Threonine 190/191 (E) in male and female mouse plantaris muscles (*n* = 4 in each, 3‐months old). The total sMyBP‐C and fMyBP‐C proteins were normalized to β‐actin expression and phosphorylation was normalized to total sMyBP‐C. M denotes the male group, and F denotes the female group. Error bars represent ±SEM and **p < 0.01 and ****p < 0.0001, male vs. female.

Our previous study demonstrated the distinctive expression of fMyBP‐C in fast‐twitch muscle fibres [12]. The fast‐twitch muscle fibres are more abundant than the slow‐twitch muscle fibres in male skeletal muscle compared to female skeletal muscle in mouse and untrained human muscle biopsy studies [17, 18]. However, a recent study using muscle biopsies of elite weightlifters reported that females of this trained group had a higher abundance of fast‐twitch fibres compared to males [19]. Therefore, we have assessed slow and fast MyBP‐C protein expressions, as well as the phosphorylation status of sMyBP‐C, in male and female mouse plantaris muscle samples. Our findings revealed a notable disparity, with male plantaris muscle exhibiting a significantly higher (75% more) fMyBP‐C expression when compared to their female counterparts (Figure 2A–C). Conversely, sMyBP‐C phosphorylation at Ser59/Ser62 and Thr190/Thr191 was significantly higher in the female samples, with increases of 4.42 and 5.08‐fold, respectively, compared to the male samples (Figure 2D,E). These results underscore the significance of fMyBP‐C in male and female muscle physiology and highlight its greater importance in the function and structure of male fast‐twitch fibres.

### 3.3 Reduced Force Generation Capacity and Disrupted Muscle Fibre Architecture in the Absence of fMyBP‐C

**FIGURE 3:**
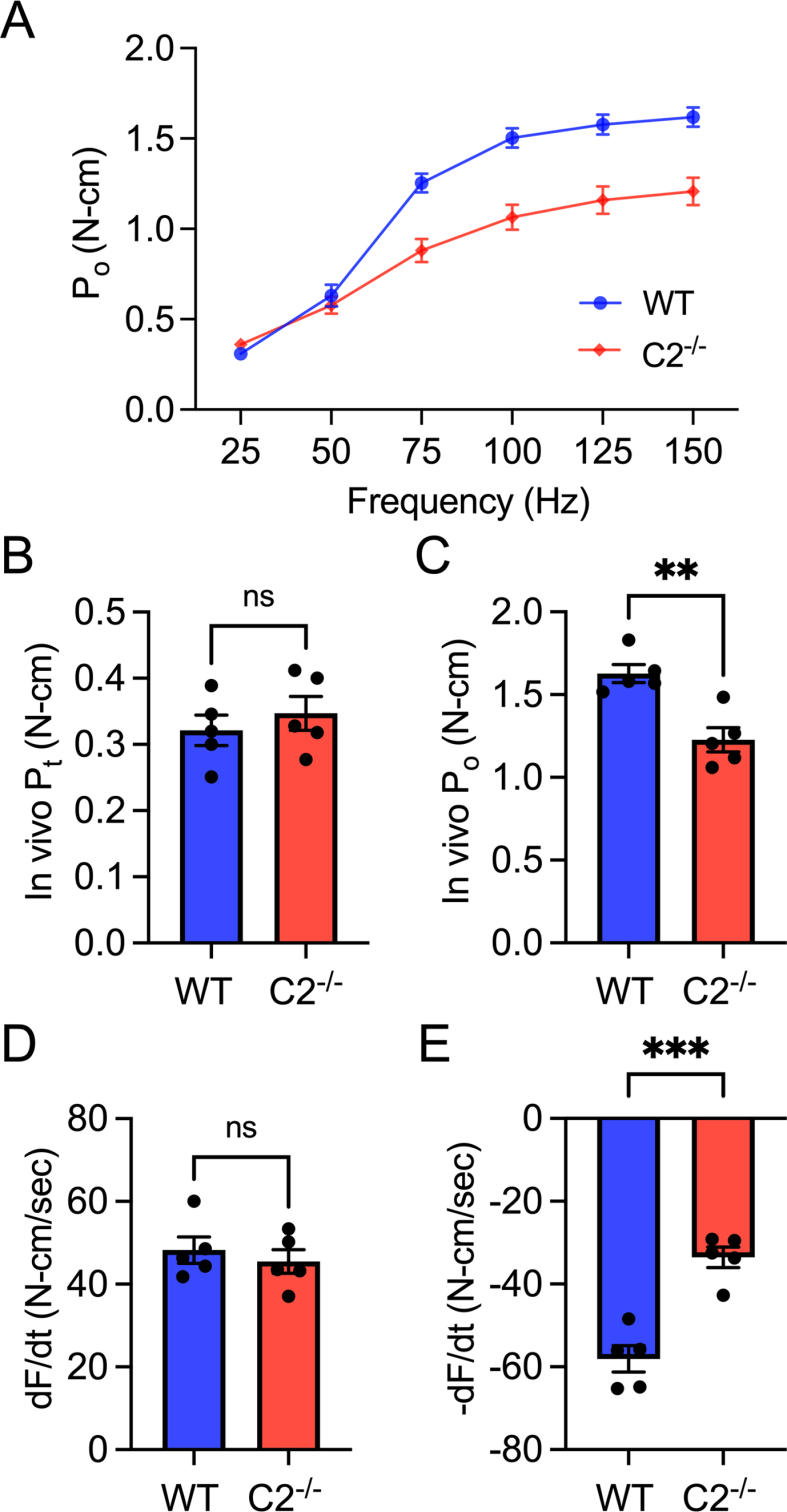
Reduced muscle function in male C2^−/−^ muscle. (A) In vivo isometric plantar flexor force generation of male WT and C2^−/−^ mice at different electrical frequencies (25–150 Hz), peak twitch force (B), peak tetanic force (C) and the rate of activation/relaxation (D and E). Mice used were aged 4–6 months. Error bars indicate ±SEM. **p < 0.01 and ***p < 0.001, C2^−/−^ vs. WT.

To examine the role of fMyBP‐C in the contractile function of skeletal muscle, we measured the in vivo isometric plantar flexor force generation in male WT and C2^−/−^ mice. As shown in the force‐frequency graph (Figure 3A), there was no difference in force generation between WT and C2^−/−^ during incomplete tetanic contractions at low electrical frequencies (25 and 50 Hz). However, at a frequency of 75 Hz and above, which induces tetanic muscle contraction, C2^−/−^ generated significantly less force than WT. Peak twitch force generation was equivalent in both groups, but peak tetanic force was significantly lower (25% less) in C2^−/−^ compared to WT (Figure 3B,C). The rate of activation during peak tetanic contraction was not different between groups, but the rate of relaxation was significantly slower (42% less) in C2^−/−^ (Figure 3D,E).

**FIGURE 4:**
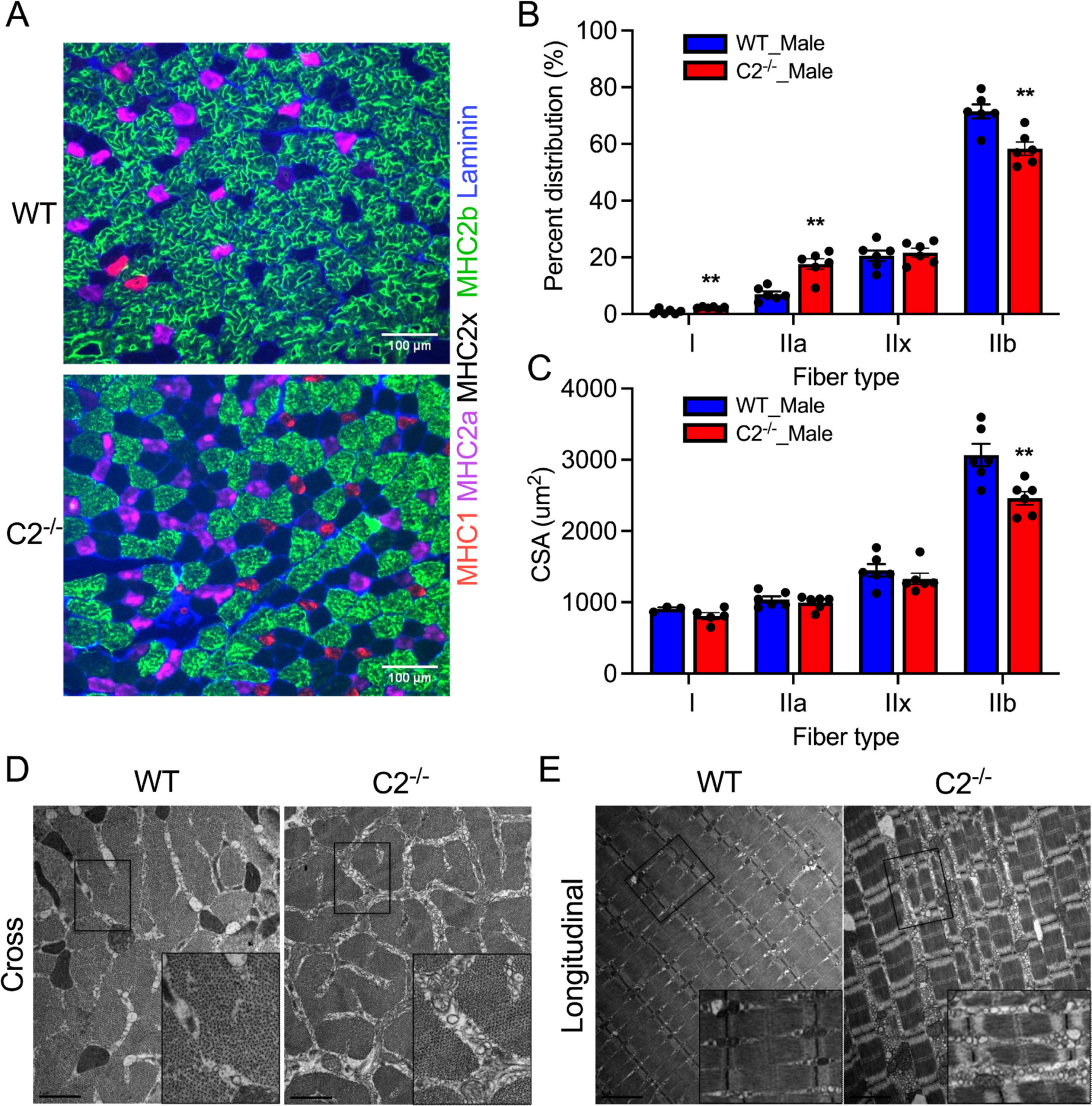
Histological maladaptation in male C2^−/−^ muscle. (A) Cross‐sectioned EDL muscles immunostained with MHC and laminin antibodies. The scale bar is 100 μm. Fast‐to‐slow fibre type switch (B) and significantly smaller size of type IIb fibres (C) in C2^−/−^ vs. WT muscle. *n* = 6 slides from three muscles in each group, aged 3–6 months. Cross (D) and longitudinal (E) sectioned EM images of EDL muscles show the accumulation of small vesicles between myofibrils of C2^−/−^. The scale bar is 1 μm (cross image) and 2 μm (longitudinal image). Error bars indicate ±SEM. **p < 0.01, C2^−/−^ vs. WT.

Histopathological adaptation of each muscle fibre defined by changes in fibre type composition, hypertrophy and atrophy in the absence of fMyBP‐C was evaluated in cross‐sectioned EDL muscle stained with myosin heavy chain (MHC) antibodies (Figure 4A). Our results showed significant muscle fibre type switching from fast to slow fibre types in C2^−/−^. The percentage of type IIb fibres significantly decreased (12.8% less) while increasing the percentages of type I and IIa fibres, 1.4% and 10.6%, respectively (Figure 4B). The average CSA of each fibre type was also measured, and a significant reduction in CSA of type IIb fibres (−20%) was observed in C2^−/−^ (Figure 4C). These results indicate that fMyBP‐C is required to maintain the number and size of fast‐twitch fibres (type IIb) in male skeletal muscle. Electron microscopy analysis of male EDL muscle demonstrated preserved overall sarcomere structure and integrity in C2^−/−^, which is consistent with what was previously shown [12]. However, we found expanded space between muscle fibres and small vesicles occupied the expanded space, possibly indicating ongoing muscle atrophy or chronic stress in the C2^−/−^ muscle (Figure 4D,E).

### 3.4 Reduced PKA‐Dependent sMyBP‐C Phosphorylation in C2^−/−^ Muscle

**FIGURE 5:**
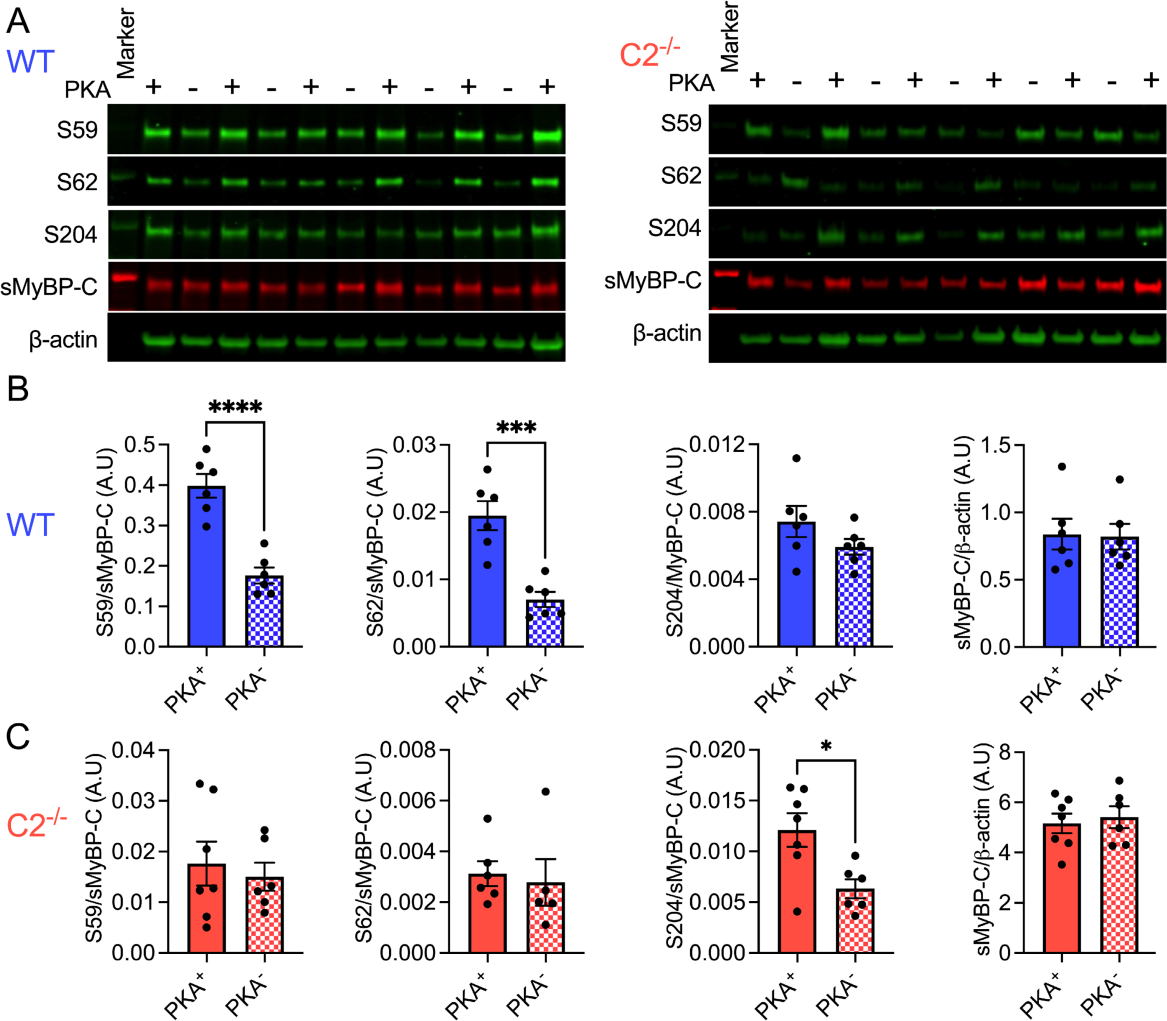
Altered PKA‐dependent sMyBP‐C phosphorylation in C2^−/−^ fibre. (A) PKA‐targeted sMyBP‐C phosphorylation was measured by Western bloting in skinned WT and C2^−/−^ EDL fibres. Quantification of sMyBP‐C phosphorylations at serine 59, serine 62, serine 204 and total sMyBP‐C in WT (B) and C2^−/−^ (C) fibres. The total sMyBP‐C protein was normalized to β‐actin expression. Mice used were aged 3–4 months. A total of 6–7 skinned fibre preparations were used. Error bars represent ±SEM and *p < 0.05, ***p < 0.001 and ****p < 0.0001, PKA+ vs. PKA−.

PKA‐dependent MyBP‐C phosphorylation is crucial in regulating muscle contraction, impacting muscle contractility and relaxation, thereby influencing overall muscle function [20]. sMyBP‐C has multiple PKA‐dependent phosphorylation sites in its N′‐terminal, and our previous study showed a compensatory increase of total sMyBP‐C protein expression in C2^−/−^ EDL muscle [12]. Therefore, we have evaluated changes in sMyBP‐C phosphorylation in skinned WT and C2^−/−^ EDL fibres after PKA treatment (Figure 5A). In WT samples, phosphorylation of sMyBP‐C at serine 59 and serine 62 was significantly increased in PKA‐treated fibres, while no changes were observed for serine 204 phosphorylation (Figure 5B). However, in C2^−/−^ mice fibres, serine 59 and serine 62 sites were not responsive to PKA treatment. Instead, phosphorylation of the serine 204 site was significantly increased after PKA treatment (Figure 5C). As expected, the total sMyBP‐C expression level was not different between PKA‐treated and non‐treated samples of WT and C2^−/−^ (Figure 5B,C).

### 3.5 Dysregulation of Critical Genes and Pathways in C2^−/−^ Muscle

To further investigate the underlying molecular mechanisms of reduced contractile functions and pathophysiological adaptations in the young male C2^−/−^ muscle, we have reanalysed male single EDL fibre RNA‐seq data (*n* = 10 in each group, GEO accession: GSE160827). We found a total of 956 DEGs (621 up and 335 down) in C2^−/−^ (Figure S3A). The 10 most upregulated and downregulated genes in C2^−/−^ fibres are listed in Figure S3B. The most upregulated genes included *Actc1*, a cardiac and developing skeletal muscle sarcomeric gene, and *Phlda3*, a positive apoptotic pathway regulator. Membrane ion and protein transporter genes, *Tm87a* and *Atp13a2*, and Wnt signaling inhibitor gene, *Apcdd1*, are among the most downregulated genes in C2^−/−^ (Figure S3C). We also found significantly disrupted biological processes (GO_BP) and molecular function (GO_MF) pathways using gene ontology analyses. Metabolic processes and membrane receptor binding pathways are upregulated, while DNA and RNA regulation pathways are downregulated (Figure S3D,E). Altogether, findings from young C2^−/−^ mice show that fMyBP‐C is an important structural and functional protein abundant in the fast‐twitch muscle fibres in male mice.

### 3.6 fMyBP‐C Is Required for Aged Muscle Function and Homeostasis

Maintaining the integrity and functionality of skeletal muscle becomes increasingly challenging with age. Preserved muscle mass and strength have been known to decrease the risk of orthopaedic injury and increase the quality of life and independence in the aged population [S4]. Literature has shown selective loss of glycolytic type II fibres with aging. Particularly, type IIb fibres (which are closely related to the IIx fibres in humans) are the most susceptible to atrophy, related to developing metabolic dysfunction in aged mouse muscle [3, 21]. In humans, type IIx fibres are particularly susceptible to atrophy with aging [22] and are the first to degenerate in Duchenne muscular dystrophy [23]. Therefore, we investigated the roles of fMyBP‐C in age‐related loss of muscle functions and structures, the rationale being that the fMyBP‐C paralog is highly expressed in the fast‐twitch muscle fibres [24].

**FIGURE 6:**
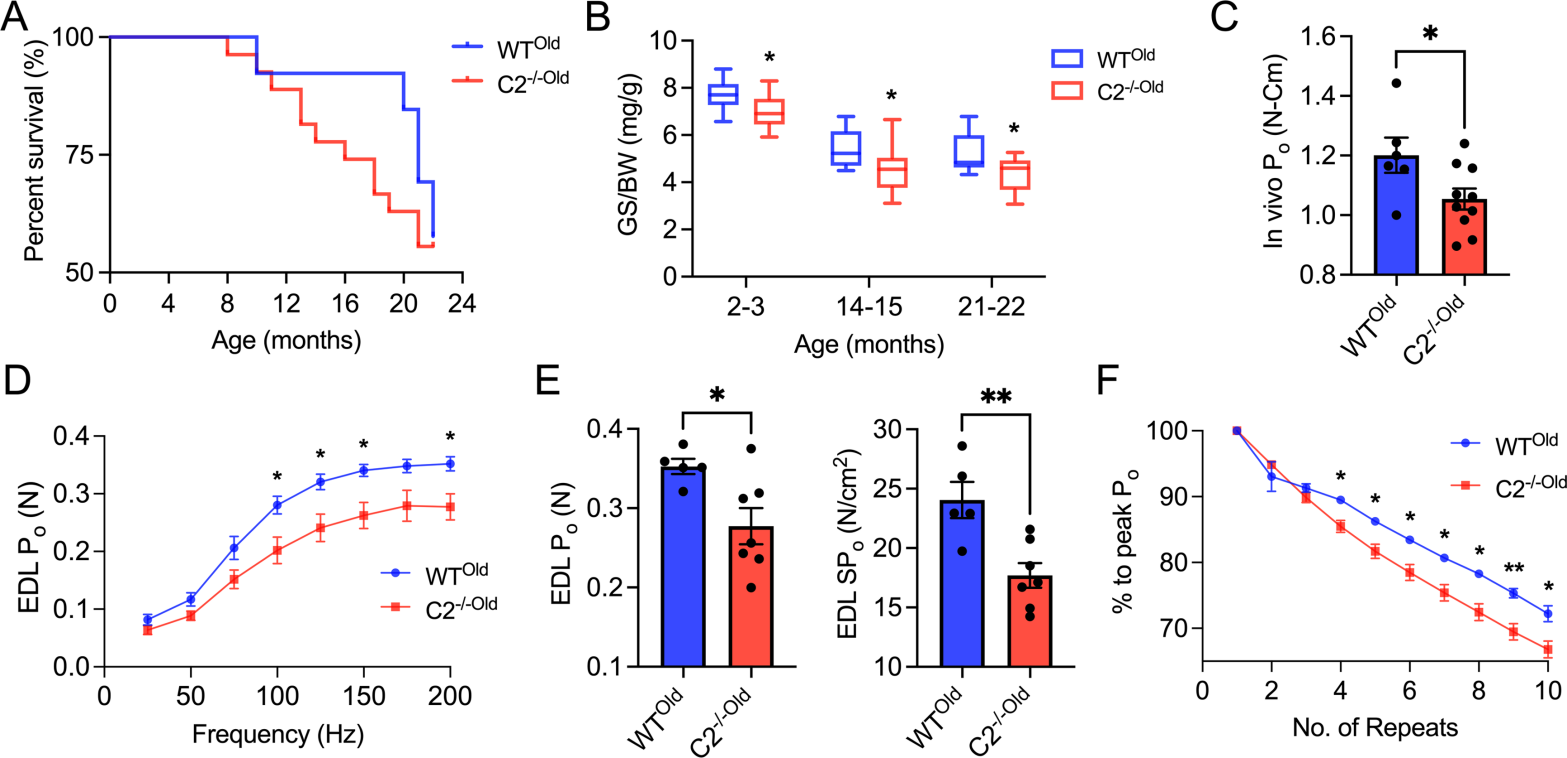
Reduced survival rate and muscle functions in C2^−/−^ mice. (A) Mouse survival rate decreased after 8 months of age in C2^−/−^ (*n* = 27) vs. WT (*n* = 13) mice. (B) Grip strength was significantly lower in C2^−/−^ (*n* = 15–16) compared with WT (*n* = 7–16) mice at all ages. (C) In vivo, peak isometric plantar flexor force generation was reduced in C2^−/−^ mice. (D) Downshift force‐frequency graph. C2^−/−^ EDL generated significantly less isometric tetanic force at mid to high electrical frequency (100–200 Hz). (E) Ex vivo EDL peak isometric tetanic (left) and specific force (right) were significantly lower in C2^−/−^ (vs. WT). (F) C2^−/−^ EDL loses significantly more force during repeated 10 isometric peak tetanic contractions. *n* = 4–10 in each group. Mice used were aged 21–22 months. Error bars represent ±SEM and *p < 0.05 and **p < 0.01, C2^−/−Old^ vs. WT^Old^.

Gross body mass and muscle weight did not differ between WT^Old^ and C2^−/−Old^ mice at 21–22 months of age (Figure S4). Although the survival rate was not significantly different between the two groups, C2^−/−Old^ mice showed a gradual decline with aging. At 20 months, 85% of WT mice were alive, while only 63% of C2^−/−Old^ mice survived (Figure 6A). Grip strength decreased with aging in both groups, but C2^−/−Old^ mice exhibited significantly lower grip strength and in vivo peak isometric plantar flexor force at 22 months compared to WT^Old^ (Figure 6B,C). To further evaluate fast‐twitch muscle functions, we isolated the EDL muscle and measured ex vivo contractile functions, which resulted in significantly reduced force generation capacity at the electrical stimulation frequency above 100 Hz in C2^−/−Old^. Compared to WT^Old^, peak isometric tetanic and specific force were significantly reduced by 21% and 26% in C2^−/−Old^, respectively. C2^−/−Old^ EDL also showed reduced fatigue resistance during repeated peak isometric tetanic contractions, showing more P_o_ loss after the fourth tetanic contraction (Figure 6D–F). We also compared the force generation capacity of EDL normalized to the muscle weight in young and old WT and C2^−/−^ mice and observed significant functional deficits both in WT^Old^ and C2^−/−Old^ mice as compared to their younger counterparts. However, the C2^−/−Old^ mice were worse off among all the groups, implicating the role of fMyBP‐C in the normal functioning of the aged muscle (Figure S5).

**FIGURE 7:**
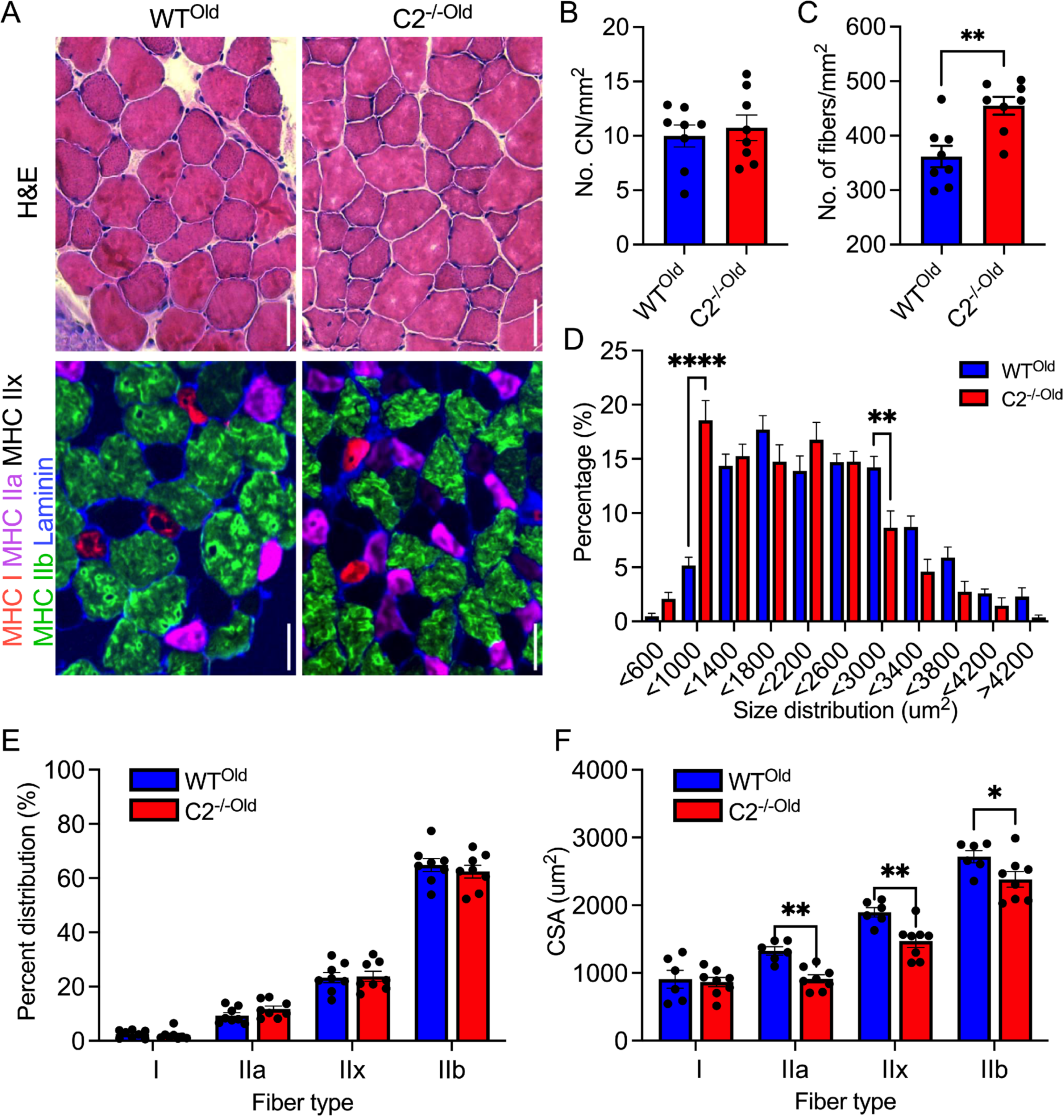
Atrophy of fast‐twitch fibres in aged C2^−/−^ EDL. (A) Representative H&E (top) and myosin heavy chain isoforms immunostained images (MHC, bottom) of WT^Old^ (left) and C2^−/−Old^ EDL. Scale bar = 50 μm. There was no difference in the numbers of central nuclei (B), but a significant increase in the total number of fibres in C2^−/−Old^ compared to WT^Old^ (C). (D) Increased percentage of small‐sized fibres while decreasing large fibres in C2^−/−Old^. (E) Preserved fibre type distributions but (F) significantly reduced the cross‐sectional area of fast‐twitch fibres (type IIa to IIb) in C2^−/−Old^. *n* = 6–8 slides from 3 to 4 mice in each group. Mice used were aged 21–22 months. Error bars mean ±SEM and *p < 0.05, **p < 0.01 and ****p < 0.0001, C2^−/−Old^ vs. WT^Old^ fibres.

Next, to evaluate histological adaptations in the aged WT and C2^−/−^ muscle, we first stained cross‐sectioned EDL samples with H&E and counted the number of central nuclei (CN), which is a hallmark of muscle regeneration after damage (Figure 7A, top). The average number of CN in each slide was not different between the two groups (Figure 2B). However, we found significantly increased fibre numbers in C2^−/−Old^ samples (Figure 7C). The muscle fibre size distribution graph shifted left in C2^−/−Old^, indicating the increased number of small‐sized muscle fibres (Figure 7C‐D). EDL slides were also immunostained with MHC antibodies and analysed for the distribution and size of each fibre type (Figure 7A, bottom). Muscle fibre type composition was not different in WT^Old^ and C2^−/−Old^ EDL muscles, but the size of fast‐twitch fibres (type IIa, IIx and IIb) was significantly reduced in C2^−/−Old^ compared to WT^Old^ (Figure 7E,F). These results emphasize that fMyBP‐C ablation results in severe muscle pathology and that it is essential for maintaining fast‐twitch fibre size and homeostasis during the aging process.

### 3.7 Disrupted Gene and Protein Expressions in Aged C2^−/−^ Muscle

**FIGURE 8:**
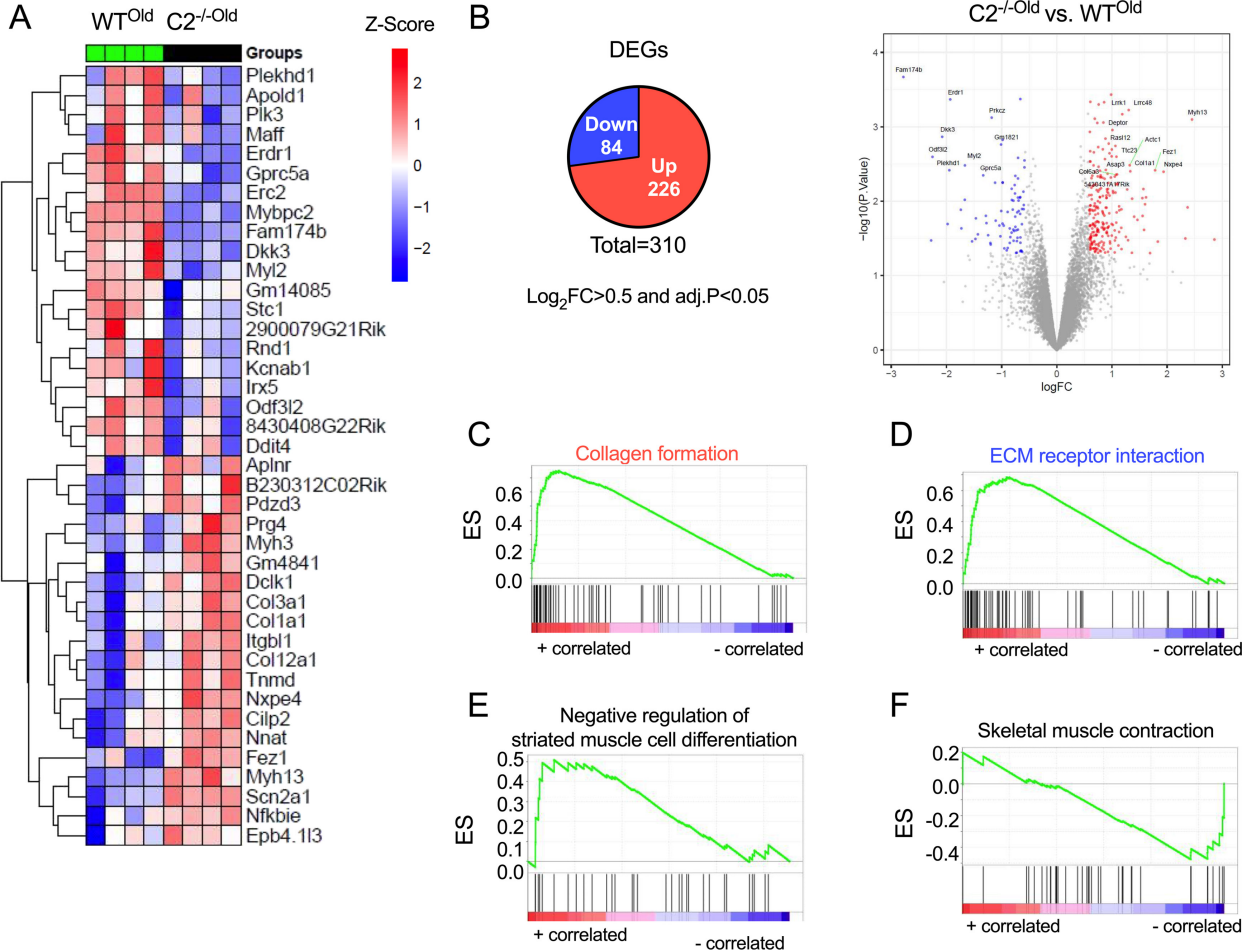
RNA sequencing reveals dysregulated genes and pathways in aged tibialis anterior C2^−/−Old^ muscle. RNA sequencing was carried out on TA muscle samples from old male C2^−/−^ and wild‐type (WT) mice, aged 21–22 months (*n* = 4, TA muscles/group), followed by differential gene expression analysis and gene set enrichment analysis. (A) Heat map of top twenty upregulated and downregulated genes, (B) Total number of differentially expressed genes based on log‐transformed fold change cut off of 0.5 and effect size threshold of adj. *p* < 0.05 (left) and volcano plot comparing DEGs in C2^−/−Old^ vs. WT^Old^ (right). (C‐F), Enrichment score graph of key molecular signatures that alter muscle structure and function in older male C2^−/−Old^ vs. WT^Old^ TA muscles.

To further explore the molecular mechanisms underlying the reduced muscle function and fibre size observed in aged C2^−/−^ muscle, we conducted an RNA expression profiling of the TA muscle using RNA‐seq analysis (GEO accession: GSE285405). The top 20 most upregulated and downregulated genes identified in aged C2^−/−^ fibres are listed in Figure 8A. Among the 310 DEGs (226 up and 84 down) in the aged samples (Figure 8B, left), the most dysregulated genes included *Actc1* (upregulated) and several genes with predicted but not yet validated for its functions. This includes *Myh13* (upregulated); MHC 13, which is predicted to enable actin filament binding activity and microfilament motor activity, *Fam174b* (downregulated), predicted to be involved in Golgi organization and expressed in hearts and *Apold1* (downregulated), predicted to enable lipid binding activity and is expressed in cardiac ventricles (Figure 8B, right). Independently, we also performed RNA sequencing of TA muscle in young mice (5–6 months of age; GEO accession: GSE302562) and compared DEGs in young and aged mice. DEGs from C2^−/−Young^ and C2^−/−Old^ mice revealed common signatures (45 upregulated genes and 14 downregulated genes), which indicate that these are the effects of the fMyBP‐C ablation (Figure S8C). In addition, there is a specific set of genes that were either upregulated or downregulated in C2^−/−Old^ mice, implicating the role of fMyBP‐C in the aging process. More importantly, functional enrichment analysis revealed that the fibrillar collagen trimer gene set was enriched in both C2^−/−Young^ and C2^−/−Old^ upregulated genes. Whereas C2^−/−Old^ mice exhibited an additional set of genes related to the assembly of collagen fibrils that were enriched (Figure S8D–F). In addition, ECM receptor interaction and negative regulation of muscle differentiation, which are linked to muscle damage and impaired regeneration, were significantly increased in C2^−/−Old^ (Figures 8C–E and S9 and S10). Concurrently, genes related to skeletal muscle contraction showed significant reductions in aged C2^−/−^ muscle (Figures 8F and S10D). We have also analysed the expression of key proteins related to muscle structure and atrophy. Notably, the expression of sMyBP‐C was significantly increased in aged C2^−/−^ muscles (Figure S11B), like the levels observed in young C2^−/−^ mice [12]. Interestingly, the expression of Myomesin‐1 (*Myom1*), which plays a crucial role in stabilizing thick filaments at the M‐band, also showed a significant increase in the aged C2^−/−^ samples compared to aged WT samples (Figure S11E). However, the protein levels of *Myh4*, *Mylpf*, *Foxo1*, *Ankrd2* and *Cryab* did not exhibit any significant differences between the two groups (Figure S11D,F–I).

## 4 Discussion

The sarcomere is a primary component in striated muscle, accounting for over 90% of muscle mass and volume. Its integrity and homeostasis are essential for maintaining muscle function and structure after muscle injury and disease [25, 26]. MyBP‐C is a key sarcomere regulatory protein, and it has been well‐known for regulating muscle contraction and relaxation as well as calcium transience [9]. fMyBP‐C is specifically expressed in fast‐twitch muscle fibres (type IIx and type IIb), which are more likely to be injured by mechanical stress and also challenged in diseased conditions such as muscular dystrophy and aging [12, 27, 28]. Previous work from our lab showed that fMyBP‐C is required for peak force generation as well as muscle regeneration after injury in fast‐twitch muscles [12]. However, there is still a lack of information on the expression profile of fMyBP‐C in injured and diseased muscles.

The present study elucidates the crucial role of fMyBP‐C in maintaining the integrity and functional capacity of fast‐twitch muscle fibres, specifically in male mouse skeletal muscle. The observed reductions in fMyBP‐C expression in both diabetic and MDX models, as well as following eccentric contraction‐induced (ECC) muscle injury, suggest that decreased fMyBP‐C levels could contribute to the impaired muscle function characteristic of these conditions [29, 30]. We found elevated levels of fMyBP‐C protein in the bloodstream after muscle injury, which emphasized the susceptibility of fMyBP‐C to be released into circulation and suggests its possible new role as a biomarker for muscle injury, much like cMyBP‐C after cardiac injury [S5]. The relative increase in sMyBP‐C in diseased models indicates a possible compensatory mechanism. However, this adaptation appears insufficient to fully maintain muscle contractility, particularly under high‐force demands. Notably, the phosphorylation profile of sMyBP‐C varies significantly in response to reversible (patho)physiological (fatigue, repeated muscle contractions) and non‐reversible (aging and metabolic or genetic disease) stressors [31].

Our findings also highlight sex‐specific variations in fMyBP‐C and sMyBP‐C expression and phosphorylation. Significant differences in muscle fibre types between males and females have been observed in mice, contributing to the differences in muscle power and endurance capacity [32]. Male plantaris muscle exhibited notably higher fMyBP‐C levels, aligning with prior studies that underscore the importance of this protein in rapid, high‐force muscle contractions typically associated with fast‐twitch muscle [12, 33]. Conversely, female muscles demonstrated significantly higher sMyBP‐C phosphorylation at multiple PKA‐dependent sites, suggesting that sMyBP‐C phosphorylation may play a more prominent role in modulating contractility and relaxation in female muscle fibres. This sex‐specific expression profile also suggests that fMyBP‐C may be especially critical for supporting muscle force and health in male muscles, particularly under high‐load conditions.

In C2^−/−^ male mice, the reduction in isometric force generation during tetanic contractions and the delayed relaxation rate imply that fMyBP‐C is indispensable for optimal contractile response and recovery. Interestingly, PKA‐dependent phosphorylation of sMyBP‐C differed between WT and C2^−/−^ muscles, with WT samples displaying increased phosphorylation at Ser59 and Ser62 after PKA treatment, whereas C2^−/−^ samples exhibited phosphorylation shifts primarily at Ser204. This altered phosphorylation pattern in the absence of fMyBP‐C suggests that the typical regulatory role of sMyBP‐C may be disrupted, contributing to the observed deficits in contractile function.

Histopathological analyses of C2^−/−^ muscle fibres reveal significant adaptations, including fibre type switching from fast (type IIb) to slower fibre types (type I and IIa), accompanied by reduced CSA in type IIb fibres. Such shifts underscore the potential involvement of fMyBP‐C in sustaining both the number and size of type IIb fibres, which are vital for rapid, forceful contractions. Muscle wasting or atrophy as a result of disease or aging is frequently associated with fibre‐type switching, and the ablation of fMyBP‐C causes this phenomenon in C2^−/−^ muscles [S6]. Electron microscopy further indicated structural irregularities in C2^−/−^ muscle, including expanded inter‐fibre spaces and vesicle accumulation, potentially reflective of atrophic changes or chronic muscle stress [34, 35].

Transcriptomic analyses revealed substantial alterations in gene expression within C2^−/−^ muscle, with notable upregulation of *Actc1*, expressed in injured and diseased muscles, and *Phlda3*, which promotes apoptosis [36, 37, 38]. Downregulated genes included *Tm87a* and *Atp13a2*, associated with ion transport, and *Apcdd1*, a Wnt signaling inhibitor. The substantial transcriptional and functional changes collectively reinforce fMyBP‐C's essential role in regulating both the normal structure and contractile properties of fast‐twitch muscle fibres in male skeletal muscle.

We also highlight the essential role of fMyBP‐C in sustaining muscle integrity and function as the animals age. A previous study has shown that in fast‐twitch muscles, the phosphorylation of sMyBP‐C did not differ between adult and old mice [24]. Hence, the phenotype observed in the aging muscle cannot be attributed to sMyBP‐C phosphorylation. In aged C2^−/−^ mice, the absence of fMyBP‐C led to significant deficits in muscle strength and endurance, accompanied by changes in muscle fibre size and molecular signaling pathways critical for muscle homeostasis compared to WT mice. Although gross body mass and muscle weight remained unchanged, C2^−/−Old^ mice demonstrated a lower survival rate, reduced grip strength, and diminished peak plantar flexor force, indicating that fMyBP‐C is vital for maintaining contractile performance in aged muscle (Figure S5A).

The decreased force generation capacity observed in ex vivo experiments at high‐frequency stimulation in C2^−/−Old^ muscles further supports the hypothesis that fMyBP‐C is indispensable for efficient muscle contraction and force output, especially in fast‐twitch fibres. These impairments in contractility were compounded by reduced fatigue resistance, as evidenced by more rapid P_o_ loss during repeated tetanic contractions (Figure 6D,F). This reduction in endurance aligns with the established notion that age‐related loss of function predominantly affects fast‐twitch, glycolytic fibres (type IIb), which are more dependent on fMyBP‐C for rapid and forceful contractions [39].

The histological analysis provided additional insights into the structural adaptations associated with the absence of fMyBP‐C in aged muscle. Although CN count, an indicator of muscle injury and regeneration, did not differ between the groups, C2^−/−Old^ muscle exhibited a higher frequency of smaller muscle fibres, as indicated by the shift in fibre size distribution. This reduction in fibre size, particularly within fast‐twitch types IIa, IIx and IIb, likely contributes to the overall decline in muscle strength and highlights the importance of fMyBP‐C in preserving fast‐twitch fibre homeostasis during aging (Figure 7). However, the reduction in fast‐twitch fibre size observed in aged C2^−/−^ mice is likely not due to fibre atrophy, but rather reflects impaired muscle growth in the absence of fMyBP‐C. This is supported by the lack of increase in muscle fibre size, specifically type IIa and IIx fibres, in aged C2^−/−^ muscles compared to young C2^−/−^ muscles (Figure S5). Furthermore, in young WT and C2^−/−^ EDL and SOL muscles, we quantified Pax7‐positive satellite cells and myonuclei and found no significant differences between the two groups. These findings suggest that satellite cell abundance and myonuclear accretion during postnatal muscle growth are not disrupted by the loss of fMyBP‐C (Figure S7). Interestingly, consistent with previous literature, aging reduced the percentage of type IIb fibres in wild‐type (WT) mice (young: 71.5% vs. old: 64.8%), but it did not further decrease the type IIb fibre distribution in C2KO mice (young: 58.4% vs. old: 62.4%). This may be due to an increased number of small‐sized fibres in C2^‐/‐^ mice with aging (Figure 7C,F).

At the molecular level, our RNA‐seq analysis of the fast‐twitch muscle in young and aged C2^−/−^ samples showed widespread transcriptional changes (Figure S8C). Functional enrichment of commonly upregulated genes indicates that the deficiency of fMyBP‐C significantly altered collagen trimer pathway genes (Figure S8D). Interestingly, genes that display upregulation only in C2^−/−Old^ (diminished levels in C2^−/−^) display enrichment of genes related to the assembly of collagen fibrils and other multimeric structures (Figure S8E,F), and the skeletal muscle purine metabolism (Supporting Information). Interestingly, a long noncoding RNA (2310015D24Rik) was downregulated in young mice and elevated in older mice. Based on the Mouse Genome Informatics database, its expression is absent in the embryonic skeletal system and is specific to the adult skeletal system. As expected, like previous observations on skeletal gene expression transformation during aging [40], pathways related to extracellular matrix organization and collagen formation were significantly altered in the aged C2^−/−^ muscle. These factors, associated with tissue repair and fibrosis, were upregulated, suggesting that the aged muscle lacking fMyBP‐C undergoes structural remodeling, driven by chronic stress or muscle damage. Conversely, key pathways for immune response, protein synthesis and contractile function, including PERK signaling and skeletal muscle contraction pathways, were downregulated, indicating a compromised ability for muscle maintenance and repair. These molecular disruptions align with the observed functional impairments and suggest that fMyBP‐C plays a critical role in maintaining molecular processes essential for preserving muscle fibre composition, size and function in aged skeletal muscle.

Interestingly, sMyBP‐C protein expression increased significantly in aged C2^−/−^ muscles, consistent with observations in young C2^−/−^ mice, and may represent an adaptive response attempting to compensate for the loss of fMyBP‐C. However, this upregulation does not seem sufficient to fully restore fast‐twitch fibre function, indicating that the unique roles of fMyBP‐C in regulating cross‐bridge kinetics and sarcomere integrity are not replaceable by sMyBP‐C. Moreover, an increased expression of Myomesin‐1 (*Myom1*), a protein stabilizing the M‐band of sarcomeres, was observed, potentially as a compensatory attempt to maintain the normal structure in the absence of fMyBP‐C (Figure S11).

## 5 Conclusion

Our findings collectively provide novel insights into the sex‐specific and molecular determinants of muscle functionality and highlight the critical role of fMyBP‐C in preserving muscle contractility and structure, especially under high‐force conditions and disease. Future studies could explore the therapeutic potential of modulating fMyBP‐C expression in muscle disorders characterized by impaired fast‐twitch fibre performance. Our results also demonstrate that fMyBP‐C is indispensable for preserving the contractile function, structural integrity and molecular homeostasis of aged fast‐twitch muscle fibres. fMyBP‐C was observed to be significantly reduced in the diseased fast‐twitch muscle. The absence of fMyBP‐C accelerates age‐related declines in muscle force production, fibre size and regenerative capacity, emphasizing its potential as a therapeutic target to mitigate muscle aging and sarcopenia. Future research will focus on interventions to enhance fMyBP‐C expression in muscle, which could help preserve muscle health, strength and independence in the aging population.

## Conflicts of Interest

The authors declare no conflicts of interest.

## Supporting information




**Table S1:** Age, sex, mouse strain and tissue samples in each experiment.
**Figure S1:** Second *Mybpc2* knockout mouse model generation. (A) Wild‐type Mybpc2 sequence targeting exons 6 and 7 using the CRISPR/Cas9 system. PAM sites for sgRNAs are highlighted in red. (B) Complete knockdown of fMyBP‐C protein was confirmed in both slow (soleus, SOL) and (C) fast (plantaris, PLN) twitch muscles of the *Mybpc2* knockout mice by western blot analysis. The total sMyBP‐C and fMyBP‐C proteins were normalized to β‐actin expression. Mice used were aged 3 months. *n* = 2 muscle samples.
**Figure S2:** Loss of fMyBP‐C after muscle injury. (A) Force‐time graph during eccentric muscle contraction (ECC) of the plantar flexor muscle. (B) Cross‐sectioned lateral gastrocnemius muscle (LGAS) stained with H&E at 7 days after ECC injury. (C) Decreased fMyBP‐C expression post‐ECC induced muscle injury. (D) ELISA assay detected elevated fMyBP‐C levels in the blood after ECC injury. One day after ECC contraction, the GAS muscle was dissected and incubated in 800‐μL PBS solution. One hundred microliters of effluent was collected at 0.5, 1.0, 3.0 and 6.0 h after incubation. (E) Coomassie‐stained gel image loaded with 10‐μL effluent. (F) Slow and fast MyBP‐C were detected in the ECC injured effluent incubated for 6.0 h. Mice used were aged 2–3 months.
**Figure S3:** RNA sequencing reveals dysregulated genes and pathways in young male C2^−/−^ EDL muscle fibre. RNA sequencing was carried out on EDL muscle samples from young male C2^−/−^ and wild‐type (WT) mice, aged 2–3 months (*n* = 10 fibres/group), followed by differential gene expression analysis and gene set enrichment analysis. (A) Total number of differentially expressed genes based on log‐transformed fold change cut‐off of 0.5 and effect size threshold of adj. *p* < 0.05. (B) Heat map of top 10 upregulated and downregulated genes and (C) volcano plot comparing DEGs in C2^−/−^ vs. WT. (D, E) Gene set enrichment analysis of DEGs revealed the top upregulated and downregulated biological processes (D) and molecular function (E) in C2^−/−^ vs. WT. EDL muscles.
**Figure S4:** Preserved body and muscle weight in C2^−/−Old^ mice. Absolute body weight (A) and normalized hindlimb muscle mass (B) by body weight at 21**–**22 months were not significantly different between WT (*n* = 6–16) and C2^−/−^ (*n* = 15–16) mice. Error bars represent ±SEM.
**Figure S5:** Reduced C2^−/−^ EDL muscle functions with aging. (A) Peak isometric tetanic force (Po) of young (4–6 months) and old (21–22 months) WT and C2^−/−^ EDL muscles. Rate of activation (B) and relaxation (C) during P_o_ generation in aged WT and C2^−/−^. *n* = 5–9 muscles, *p < 0.05, **p < 0.01, ***p < 0.001 and ****p < 0.0001, comparing each group to every other group.
**Figure S6:** Impaired fast‐twitch fibre growth with aging in C2^−/−^ EDL. (A) Lack of increase in CSA of fast‐twitch fibres (type IIa and IIx) in C2^−/−^ with aging. *n* = 6–8 slides from 3 to 4 mice in young WT and C2^−/−^ (3–6 months, male) and old WT and C2^−/−^ (21–22 months, male) mice. Error bars mean ± SEM and **p < 0.05, ***p < 0.001 and ****p < 0.0001, comparing each group to every other group.
**Figure S7:** Preserved satellite cell and myonuclei numbers in young C2−/− muscle. Cross‐sectioned EDL and soleus (SOL) muscles were immune‐stained with Pax7 (satellite cells) and laminin (basal lamina), with nuclei counterstained using DAPI. (A) Representative image of SOL muscle at 20× magnification. Scale bar = 25 μm. Quantification of satellite cell numbers per mm2 in EDL (B) and SOL (C) muscles revealed no significant differences between WT and C2−/−groups. The average number of myonuclei per fibre, calculated from 20 fibres per slide, also showed no significant difference between WT and C2−/− EDL (D) and SOL (E) muscles. Data represent 3–4 slides from two mice per group (one male and one female, aged 4–5 months). Error bars indicate mean ± SEM.
**Figure S8:** Comparative analysis of young and old fast‐twitch muscle transcriptome in the absence of fMyBP‐C. RNA sequencing was carried out on TA muscle samples from young male C2^−/−^ (*n* = 3) and wild‐type (WT) mice (*n* = 4), followed by differential gene expression analysis and gene set enrichment analysis. The RNA sequencing data from the young (3–5 months) and old (21–22 months) TA muscles were compared to identify the C2^−/−^ and age‐specific alterations of the muscle transcriptome. (A) Total number of differentially expressed genes based on log‐transformed fold change cut‐off of 0.5 and effect size threshold of adj. *p* < 0.05. (B) Volcano plot comparing DEGs in C2^−/−Young^ vs. WT^Young^. (C) Venn diagram displaying the number of genes that display C2^−/−^ and age‐specific dysregulation in TA muscle. (D) Genes upregulated in both C2^−/−Young^ and C2^−/−Old^ mice (Common_Up), uniquely upregulated in C2^−/−Old^ mice (C2^−/−Old^ _Up) and genes downregulated in C2^−/−Young^ but upregulated in C2^−/−Old^ mice (C2^−/−^ _Down_ C2^−/− Old^ _Up) were analysed using Metascape. Enrichment of extracellular matrix and collagen assembly pathways was observed in C2^−/−^ mice. Numbers on each bar indicate the ratio of enriched genes to the total number of upregulated genes (enriched genes/total upregulated genes). Gene Set Enrichment Analysis (GSEA) was performed using GOBP, KEGG and REACTOME databases. Heat map of upregulated core genes in young C2^−/−^ (E) and C2^−/−Old^ (F) selected by their enrichment in REACTOME: Assembly of collagen fibrils and other multimeric structures and REACTOME: Collagen chain trimerization.
**Figure S9:** Heat map of upregulated core enrichment genes in aged C2^−/−^ selected by GSEA. (A) KEGC_ECM receptor interaction. (B) GOBP_Phagocytosis. (C) GOBP_Negative regulation of striated muscle cell differentiation. *n* = 4 TA samples. Mice used were aged 21–22 months.
**Figure S10:** Heat map of downregulated core enrichment genes in aged C2^−/−^ selected by GSEA. (A) GOBP_Positive regulation of immunoglobulin production. (B) KEGG_Ribosome. (C) REACTOME_PERK regulates gene expression. (D) GOBP_Skeletal muscle contraction. *n* = 4 TA samples. Mice used were aged 21–22 months.
**Figure S11:** Expression of key sarcomere structure and muscle atrophy‐related proteins in aged WT and C2^−/−^ TA muscle. Western blot images (A) and quantification of sarcomere protein (sMyBP‐C (B), fMyBP‐C (C), Myh4 (D), Myom1 (E), Mylpf (F) and atrophy‐related proteins (Foxo1 (G), Ankrd2 (H), Cryab (I) expressions normalized to β‐actin expression. Error bars mean ± SEM and **p < 0.01 and ****p < 0.0001, C2^−/−Old^ vs. WT^Old^. *n* = 5 mice muscle samples. Mice used were aged 21–22 months.

**Data S1:** Supplementary information.

**Data S2:** Supplementary information.

## Data Availability

RNA‐Seq data was deposited in NCBI's Gene Expression Omnibus database (Access No. GSE160827, GSE285405 and GSE302562).
