# Supplementary material for: Fast Myosin Binding Protein‐C Is a Vital Regulator in Young and Aged Fast Skeletal Muscle Homeostasis
Source: J Cachexia Sarcopenia Muscle. 2025 Nov 13;16(6):e70106. doi: 10.1002/jcsm.70106 (PMC12612635; doi:10.1002/jcsm.70106)
Supplement: Supplementary file 1 — Table S1: Age, sex, mouse strain and tissue samples in each experiment. Figure S1: Second Mybpc2 knockout mouse model generation. (A) Wild‐type Mybpc2 sequence targeting exons 6 and 7 using the CRISPR/Cas9 system. PAM sites for sgRNAs are highlighted in red. (B) Complete knockdown of fMyBP‐C protein was confirmed in both slow (soleus, SOL) and (C) fast (plantaris, PLN) twitch muscles of the Mybpc2 knockout mice by western blot analysis. The total sMyBP‐C and fMyBP‐C proteins were normalized to β‐actin expression. Mice used were aged 3 months. n = 2 muscle samples. Figure S2: Loss of fMyBP‐C after muscle injury. (A) Force‐time graph during eccentric muscle contraction (ECC) of the plantar flexor muscle. (B) Cross‐sectioned lateral gastrocnemius muscle (LGAS) stained with H&E at 7 days after ECC injury. (C) Decreased fMyBP‐C expression post‐ECC induced muscle injury. (D) ELISA assay detected elevated fMyBP‐C levels in the blood after ECC injury. One day after ECC contraction, the GAS muscle was dissected and incubated in 800‐μL PBS solution. One hundred microliters of effluent was collected at 0.5, 1.0, 3.0 and 6.0 h after incubation. (E) Coomassie‐stained gel image loaded with 10‐μL effluent. (F) Slow and fast MyBP‐C were detected in the ECC injured effluent incubated for 6.0 h. Mice used were aged 2–3 months. Figure S3: RNA sequencing reveals dysregulated genes and pathways in young male C2−/− EDL muscle fibre. RNA sequencing was carried out on EDL muscle samples from young male C2−/− and wild‐type (WT) mice, aged 2–3 months (n = 10 fibres/group), followed by differential gene expression analysis and gene set enrichment analysis. (A) Total number of differentially expressed genes based on log‐transformed fold change cut‐off of 0.5 and effect size threshold of adj. p < 0.05. (B) Heat map of top 10 upregulated and downregulated genes and (C) volcano plot comparing DEGs in C2−/− vs. WT. (D, E) Gene set enrichment analysis of DEGs revealed the top upregulated and downre [file JCSM-16-e70106-s003.docx]

**Supplementary Tables and Figures**

**Fast Myosin Binding Protein-C is a Vital Regulator in Young and Aged Fast Skeletal Muscle Homeostasis**

Akhil Baby et al.,

Corresponding author: Taejeong Song, tjsong@arizona.edu

The PDF file includes: **Table S1** and Figures S1 to S**11**

**Table S1. Age, sex, mouse strain, and tissue samples in each experiment.**

| **Experiments** | **Muscle** | **Gender** | **Age**  **(months)** | **Mouse model/ strain** | **Reference Figure** |
| --- | --- | --- | --- | --- | --- |
| Western blot | TA | Male and female | 3-7 | WT, MDX and db/db (C57BL/6) | 1 |
| In vivo muscle function | Plantar flexor | Male | 4-6 | WT and C2KO (C57BL/6) | 2 |
| Histology | EDL | Male | 3-6 | WT and C2KO (FVBN) | 2 |
| Western blot | EDL | Male | 3-4 | WT and C2KO (C57BL/6) | 3 |
| RNAseq | EDL | Male | 2-3 | WT and C2KO (FVBN) | 4 |
| In vivo and ex vivo muscle functions | Plantar flexor and EDL | Male | 21-22 | WT and C2KO (FVBN) | 5 |
| Histology | EDL | Male | 21-22 | WT and C2KO (FVBN) | 6 |
| RNA Sequencing | TA | Male | 21-22 | WT and C2KO (FVBN) | 7 |

Figure S1

**Figure S1.** **Second *Mybpc2* knockout mouse model generation.** A. Wild-type Mybpc2 sequence targeting exons 6 and 7 using **the** CRISPR/Cas9 system. PAM sites for sgRNAs are highlighted in red. B. Complete knockdown of fMyBP-C protein was confirmed in both slow (soleus, SOL) and **C.** fast (plantaris, PLN) twitch muscles of the *Mybpc2* knockout mice by western blot analysis. **The total sMyBP-C and fMyBP-C proteins were normalized to β-actin expression.** **Mice used were aged 3 months.** n = 2 muscle samples.

Figure S2


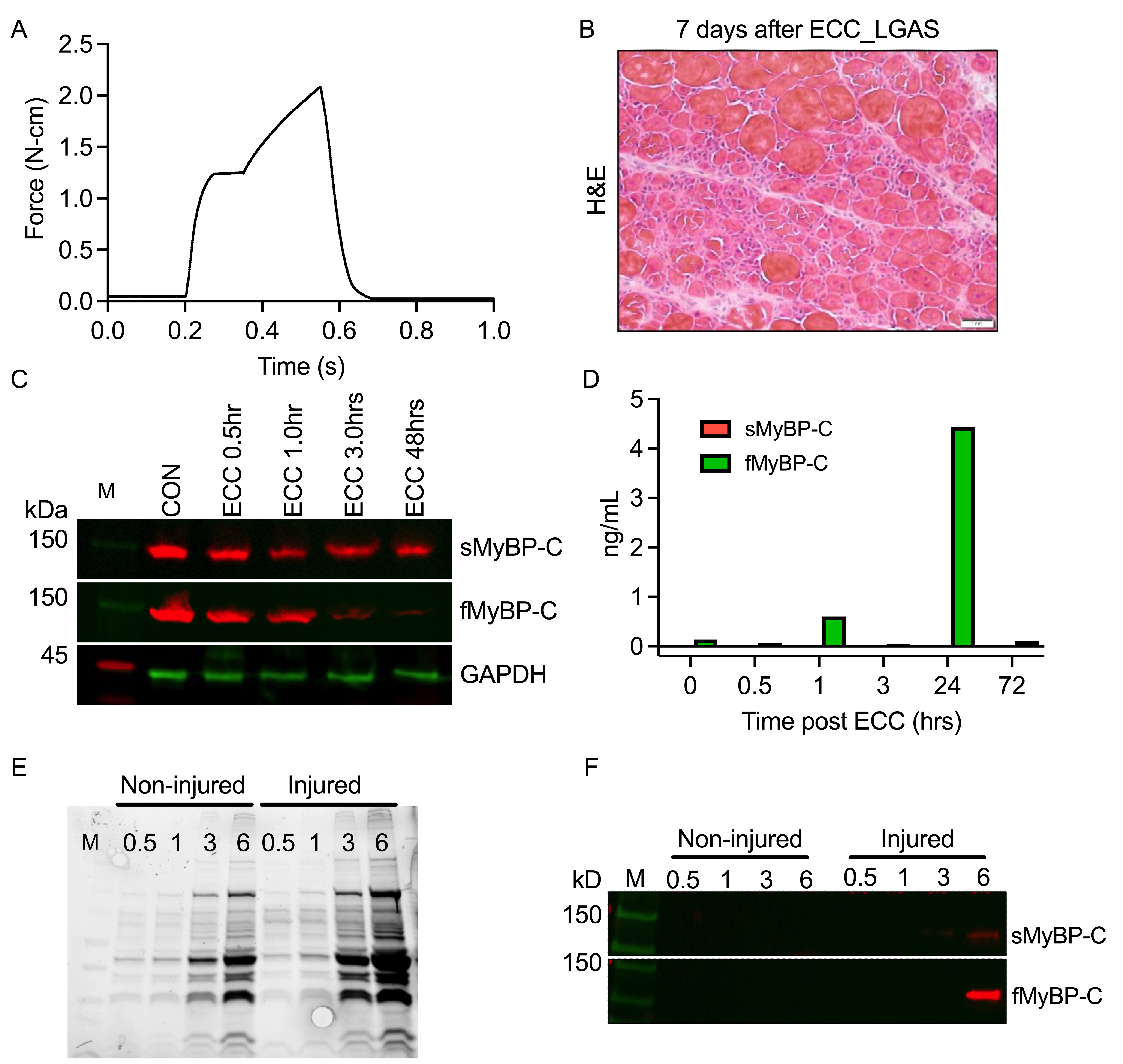


**Figure S2. Loss of fMyBP-C after muscle injury.** (A) Force-time graph during eccentric muscle contraction (ECC) of the plantar flexor muscle. (B) Cross-sectioned lateral gastrocnemius muscle (LGAS) stained with H&E at 7 days after ECC injury. (C) Decreased fMyBP-C expression post-ECC induced muscle injury. (D) ELISA assay detected elevated fMyBP-C levels in the blood after ECC injury. One day after ECC contraction, the GAS muscle was dissected and incubated in 800uL PBS solution. 100uL of effluent was collected at 0.5, 1.0, 3.0, and 6.0 hours after incubation. (E) Coomassie-stained gel image loaded with 10uL effluent. (F) Slow and fast MyBP-C were detected in the ECC injured effluent incubated for 6.0 hours. **Mice used were aged 2-3 months.**

Figure S3





**Figure S3.** **RNA sequencing reveals dysregulated genes and pathways in young male C2^-/-^ EDL muscle fiber**. RNA Sequencing was carried out on EDL muscle samples from young male C2^-/-^ and wild-type (WT) mice**, aged 2-3 months** (n = 10 **fibers**/group), followed by differential gene expression analysis and gene set enrichment analysis. (A) Total number of differentially expressed genes based on log-transformed fold change cut off of 0.5 and effect size threshold of adj. *p*<0.05. (B) Heatmap of top ten up- and down-regulated genes, and (C) Volcano plot comparing DEGs in C2^-/-^ vs. WT. (D-E), Gene set enrichment analysis of DEGs revealed the top up- and down-regulated biological processes (D) and molecular function (E) in C2^-/-^ vs. WT. EDL muscles.

Figure S4

**Figure S4. Preserved body and muscle weight in C2^-/-Old^ mice.** Absolute body weight (A) and normalized hindlimb muscle mass (B) by body weight at **21~**22 months were not significantly different between WT (n = 6-16) and C2^-/-^ (n = 15-16) mice. Error bars represent ± SEM.

Figure S5


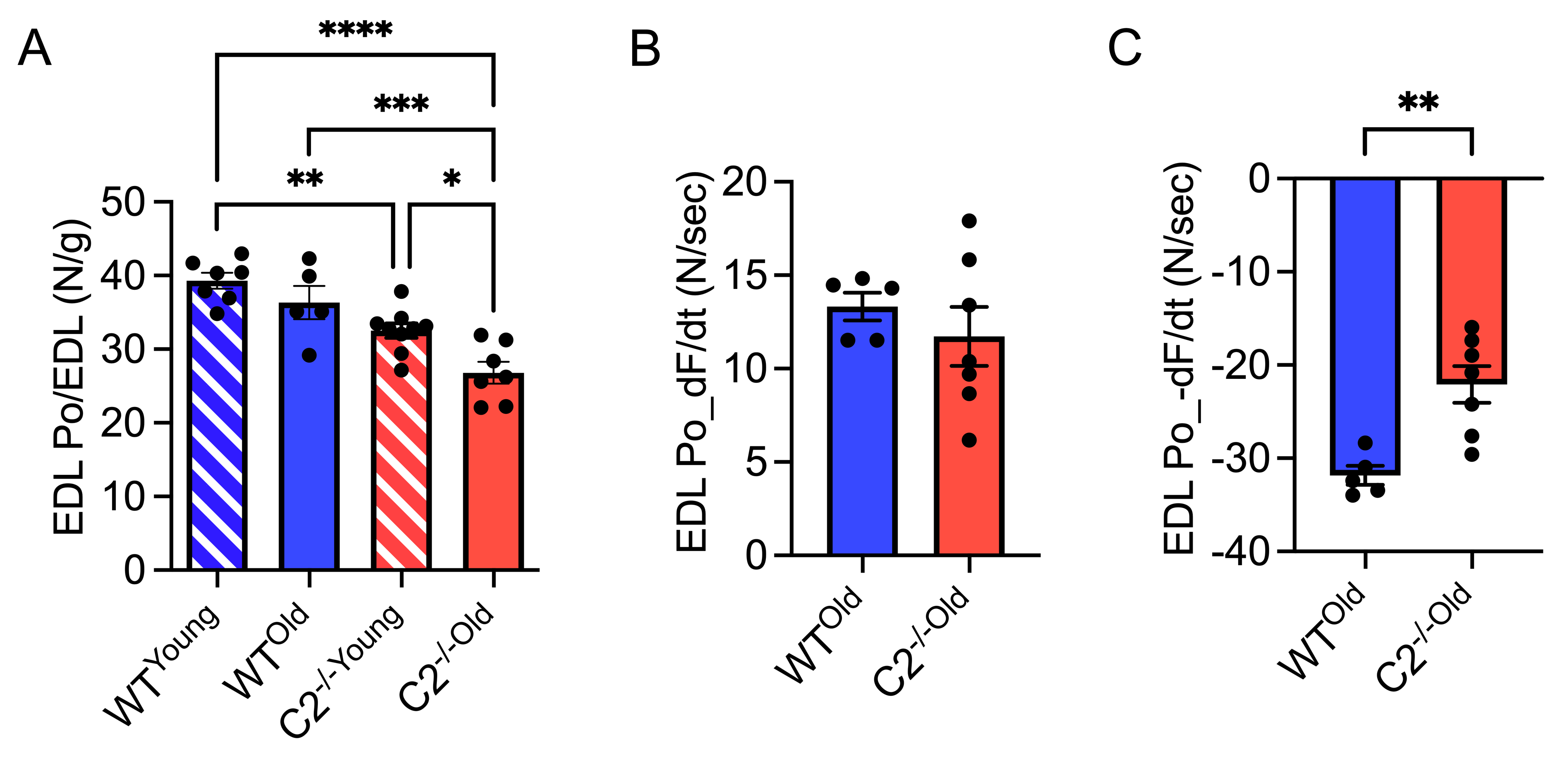


**Figure S5. Reduced C2^-/-^ EDL muscle functions with aging.** (A) Peak isometric tetanic force (Po) of young **(4-6 months)** and old **(21-22 months)** WT and C2^-/-^ EDL muscles. Rate of activation (B) and relaxation (C) during P_o_ generation in aged WT and C2^-/-^. n = 5-9 muscles, **p*<0.05, ***p*<0.01, ****p*<0.001, and *****p*<0.0001, comparing each group to every other group.

Figure S6


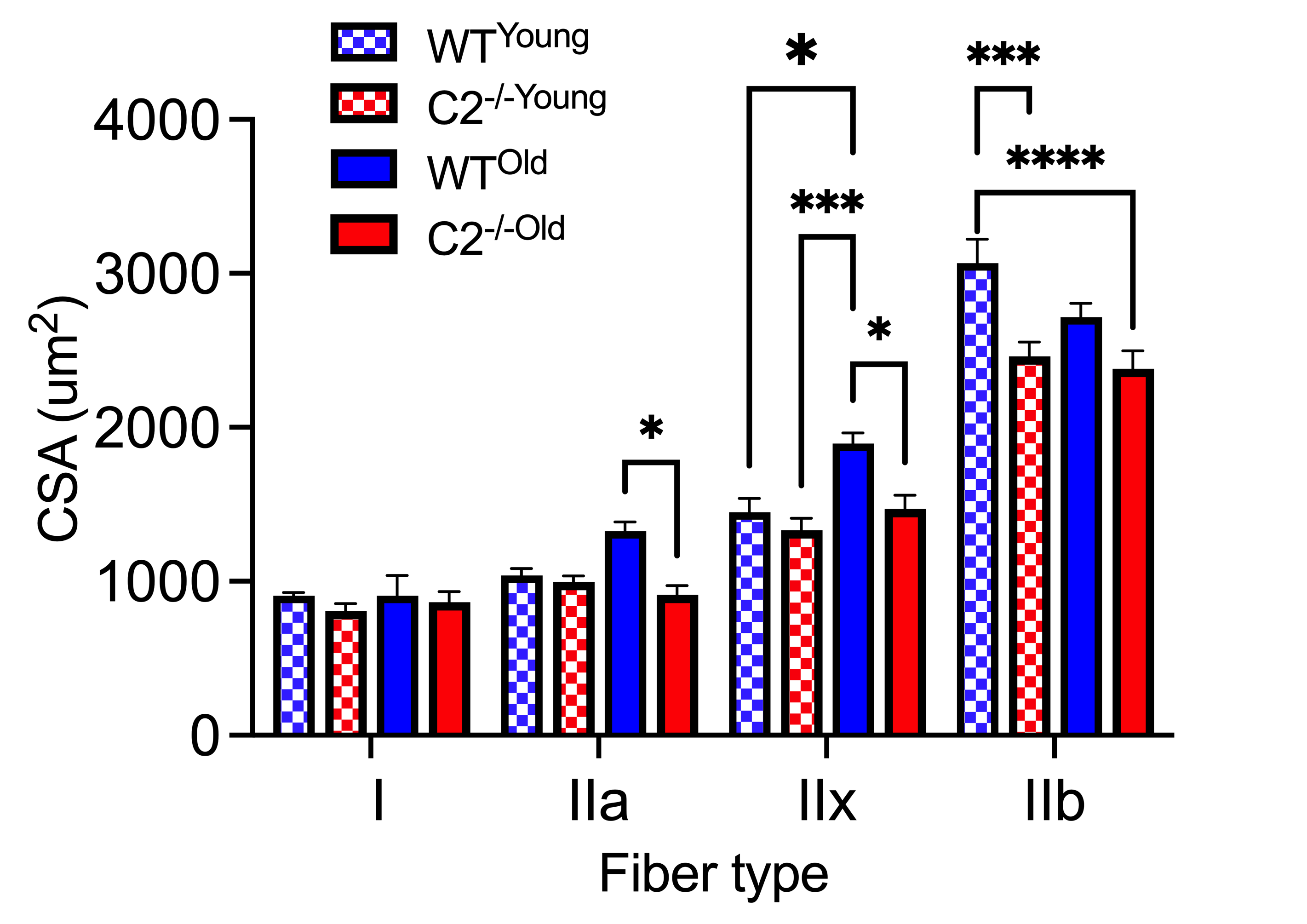


**Figure S6. Impaired fast-twitch fiber growth with aging in C2^-/-^ EDL. (A) Lack of increase in CSA of fast-twitch fibers (type IIa and IIx) in C2^-/-^ with aging. n = 6-8 slides from 3-4 mice in young WT and C2^-/-^ (3-6 months, male) and old WT and C2^-/-^ (21-22 months, male) mice. Error bars mean ± SEM and ***p*<0.05, ****p*<0.001, and *****p*<0.0001,** comparing each group to every other group.

Figure S7

**
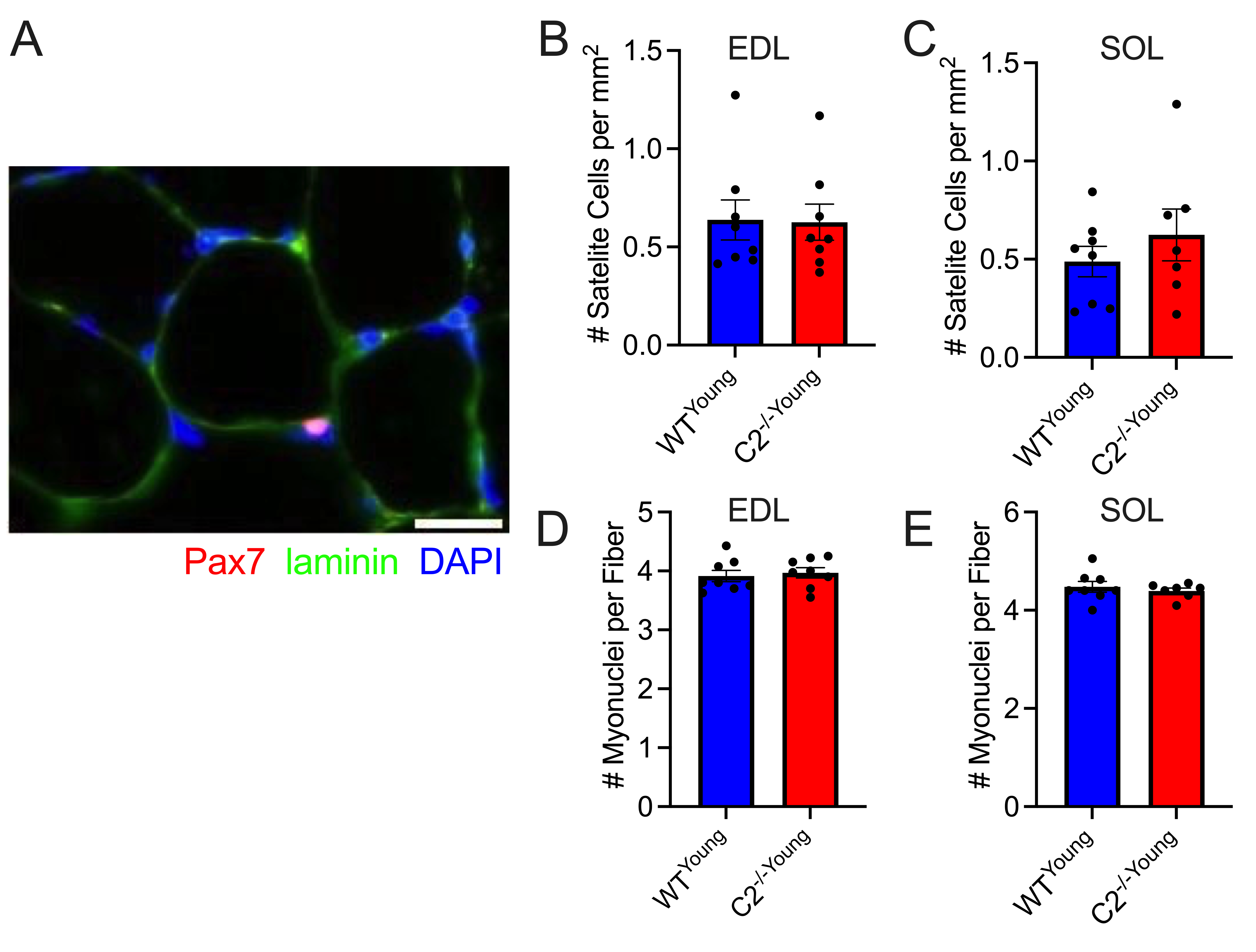
**

**Figure S7. Preserved satellite cell and myonuclei numbers in young C2^-/-^ muscle. Cross-sectioned EDL and soleus (SOL) muscles were immune-stained with Pax7 (satellite cells) and laminin (basal lamina), with nuclei counterstained using DAPI. A. Representative image of SOL muscle at 20X magnification. Scale bar = 25 μm. Quantification of satellite cell numbers per mm² in EDL (B) and SOL (C) muscles revealed no significant differences between WT and C2^-/-^groups. The average number of myonuclei per fiber, calculated from twenty fibers per slide, also showed no significant difference between WT and C2^-/-^ EDL (D) and SOL (E) muscles. Data represent 3–4 slides from two mice per group (one male and one female, aged 4–5 months). Error bars indicate mean ± SEM.**

Figure S8
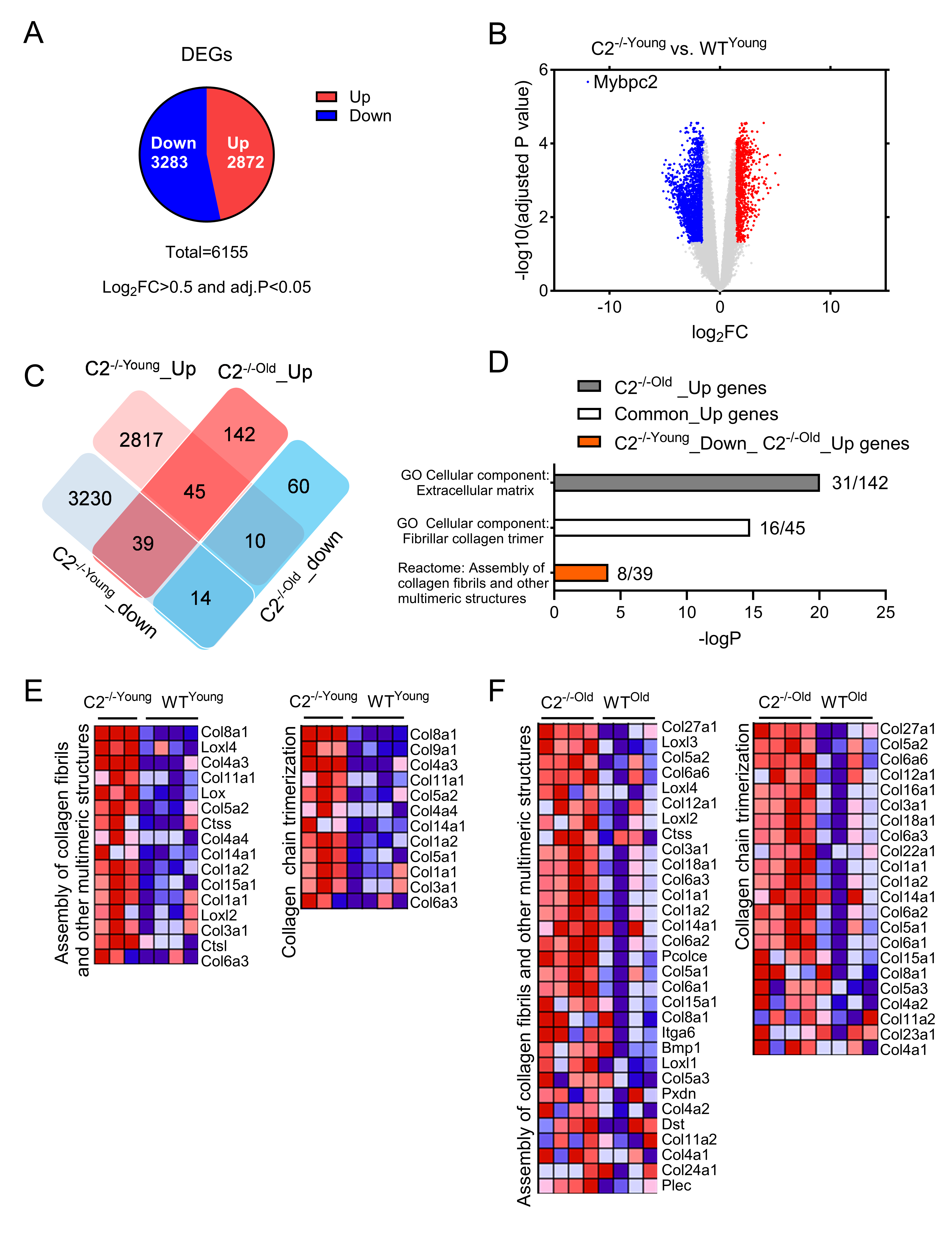


**Figure S8.** **Comparative analysis of young and old fast-twitch muscle transcriptome in the absence of fMyBP-C.** RNA Sequencing was carried out on TA muscle samples from young male C2^-/-^ (n = 3) and wild-type (WT) mice (n = 4), followed by differential gene expression analysis and gene set enrichment analysis. The RNA sequencing data from the young **(3-5 months)** and old **(21-22 months)** TA muscles were compared to identify the C2^-/-^ and age-specific alterations of **the** muscle transcriptome. (A) Total number of differentially expressed genes based on log-transformed fold change cut off of 0.5 and effect size threshold of adj. *p*<0.05. (B) Volcano plot comparing DEGs in C2^-/-Young^ vs. WT^Young^. (C) Venn diagram displaying the number of genes that display C2^-/-^ and age-specific dysregulation in TA muscle. **(D) Genes upregulated in both C2^⁻/⁻Young^ and C2^⁻/⁻ Old^ mice (Common_Up), uniquely upregulated in C2^⁻/⁻ Old^ mice (C2^⁻/⁻ Old^ _Up), and genes downregulated in C2^⁻/⁻Young^ but upregulated in C2^⁻/⁻ Old^ mice (C2^⁻/⁻^ _Down_ C2^⁻/⁻ Old^ _Up) were analyzed using Metascape. Enrichment of extracellular matrix and collagen assembly pathways was observed in C2^⁻/⁻^ mice. Numbers on each bar indicate the ratio of enriched genes to the total number of upregulated genes (enriched genes / total upregulated genes). Gene Set Enrichment Analysis (GSEA) was performed using GOBP, KEGG, and REACTOME databases. H**eat map of upregulated core genes in young C2^-/-^ **(E) and** C2^-/-Old^ (F) selected by their enrichment in REACTOME: Assembly of collagen fibrils and other multimeric structures and REACTOME: Collagen chain trimerization.

Figure S**9**


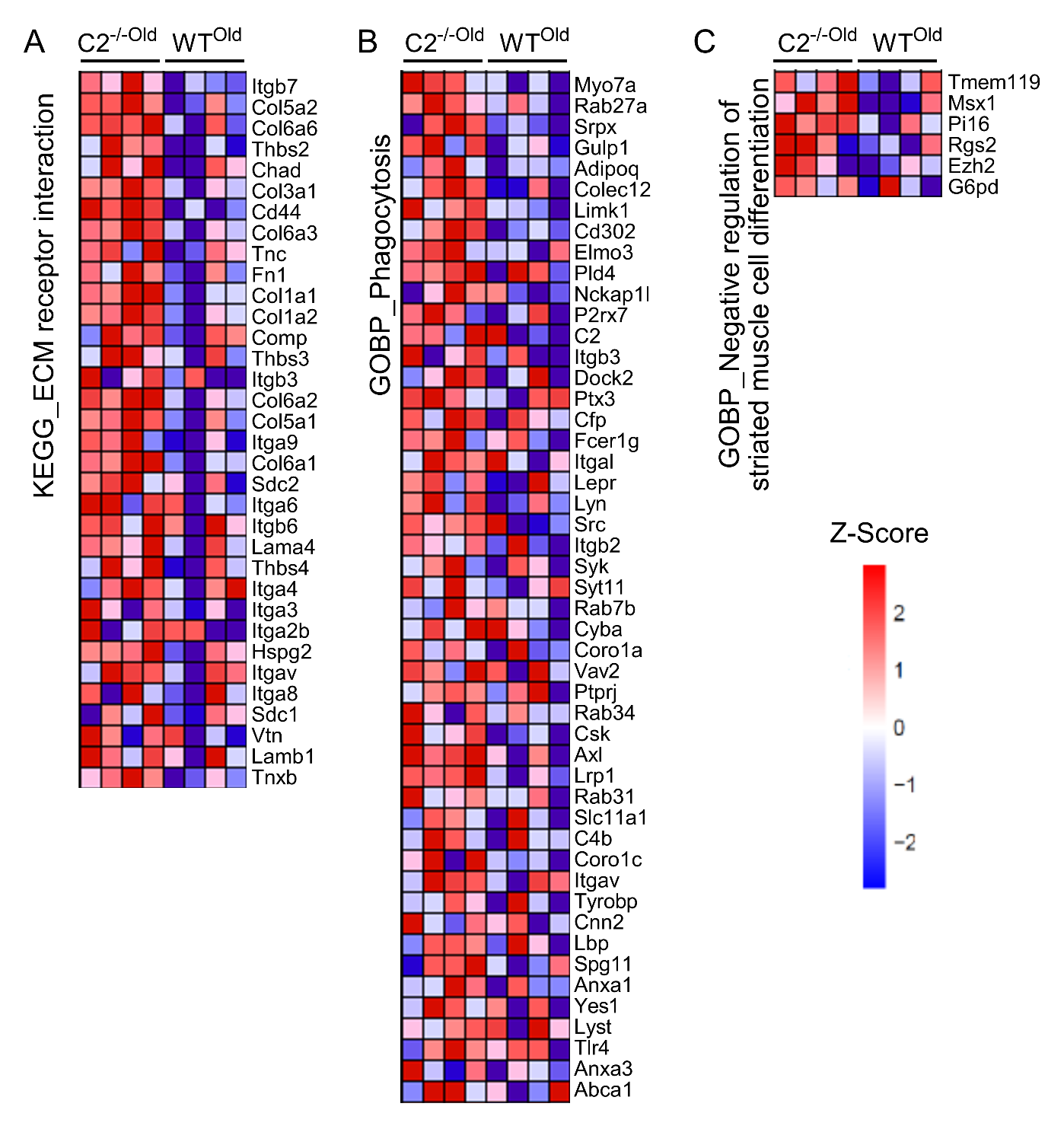


**Figure S9. Heat map of upregulated core enrichment genes in aged C2^-/-^ selected by GSEA.** (A) KEGC_ECM receptor interaction. (B) GOBP_Phagocytosis. (C) GOBP_Negative regulation of striated muscle cell differentiation. n = 4 TA samples. **Mice used were aged 21-22 months.**

Figure S**10**


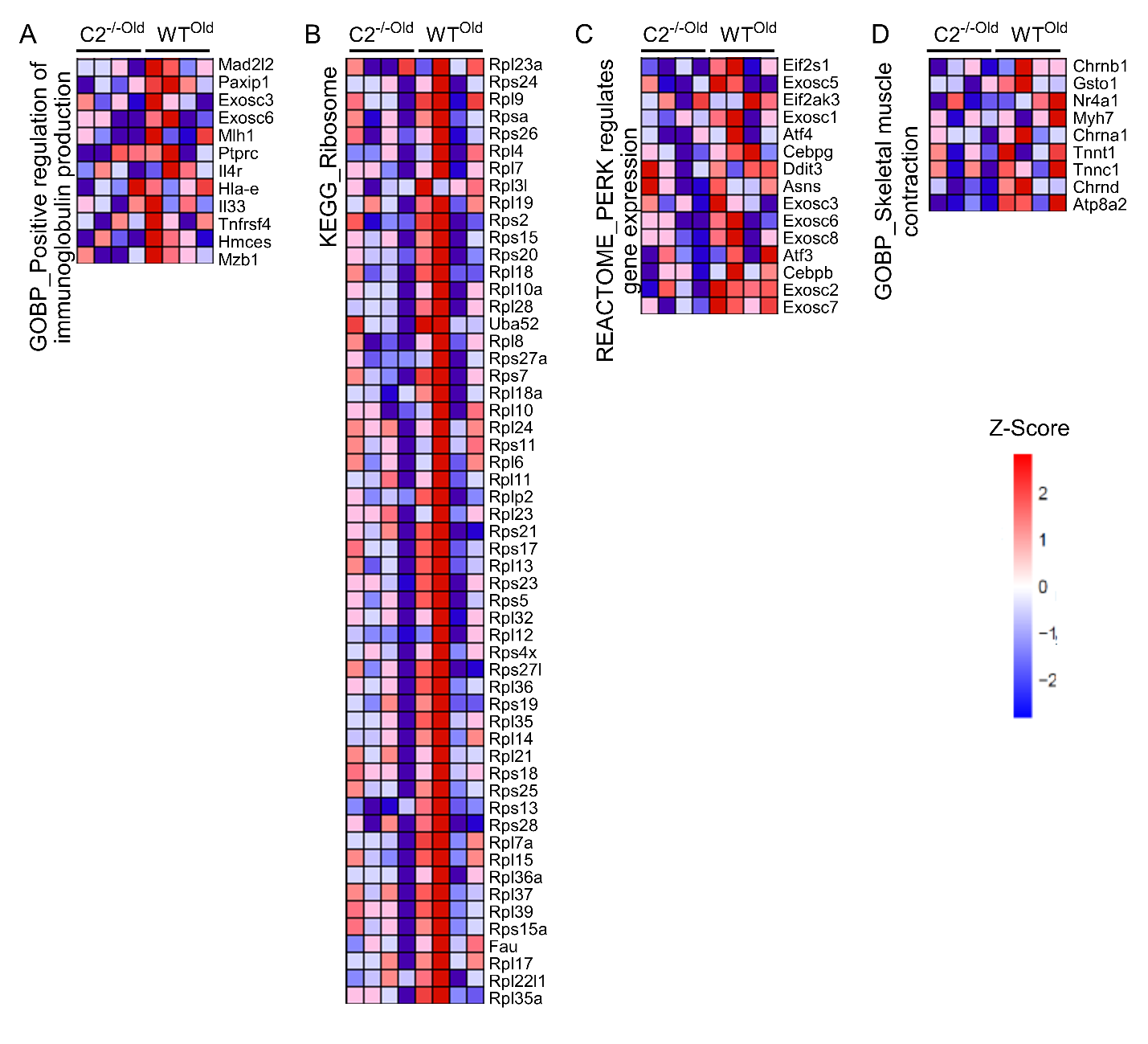


**Figure S10. Heat map of downregulated core enrichment genes in aged C2^-/-^ selected by GSEA.** (A) GOBP_Positive regulation of immunoglobulin production. (B) KEGG_Ribosome. (C) REACTOME_PERK regulates gene expression. (D) GOBP_Skeletal muscle contraction. n = 4 TA samples. **Mice used were aged 21-22 months.**

Figure S**11**


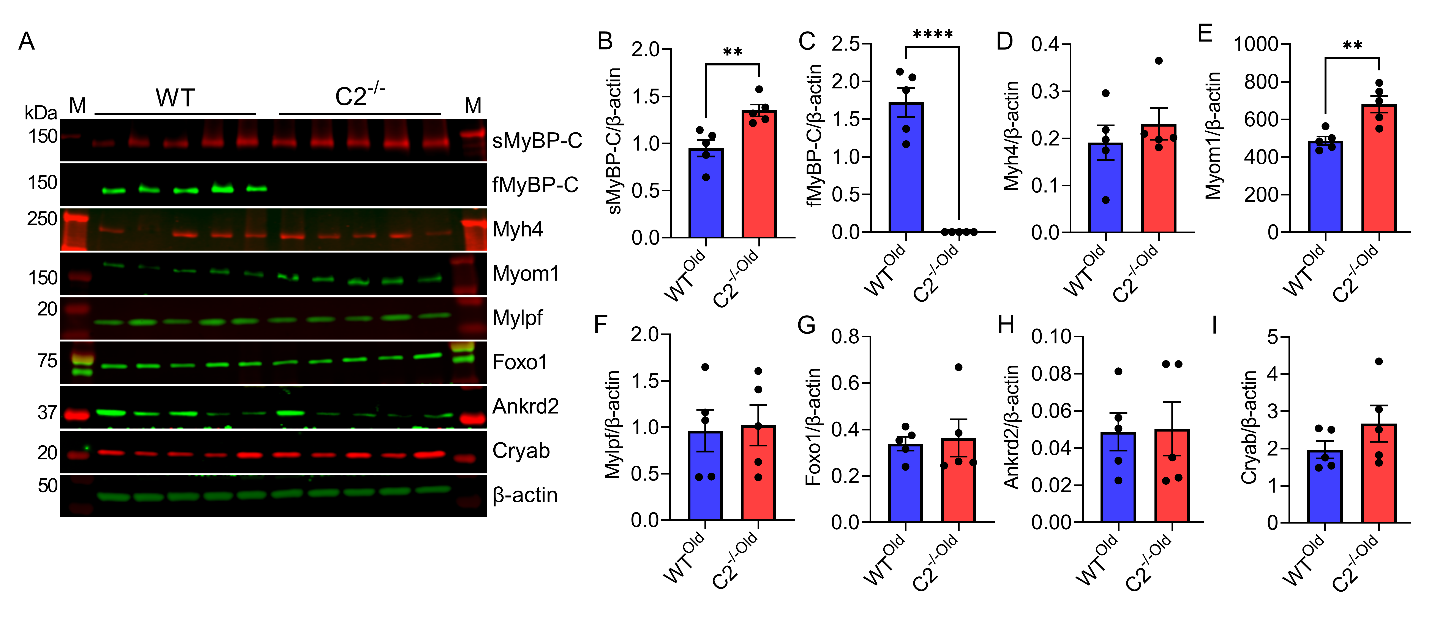


**Figure S11. Expression of key sarcomere structure and muscle atrophy-related proteins in aged WT and C2^-/-^ TA muscle.** Western blot images (A) and quantification of sarcomere protein (sMyBP-C (B), fMyBP-C (C), Myh4 (D), Myom1 (E), Mylpf (F) and atrophy-related proteins (Foxo1 (G), Ankrd2 (H), Cryab (I) expressions **normalized to β-actin expression**. Error bars mean ± SEM and ***p*<0.01 and *****p*<0.0001, C2^-/-Old^ vs. WT^Old^. n = 5 mice muscle samples. **Mice used were aged 21-22 months.**
