# Supplementary material for: Fast Myosin Binding Protein‐C Is a Vital Regulator in Young and Aged Fast Skeletal Muscle Homeostasis
Source: J Cachexia Sarcopenia Muscle. 2025 Nov 13;16(6):e70106. doi: 10.1002/jcsm.70106 (PMC12612635; doi:10.1002/jcsm.70106)
Supplement: Supplementary file 2 — Data S1: Supplementary information. [file JCSM-16-e70106-s001.docx]

**Supplementary Reference**

S1. Song T, Manoharan P, Millay DP, Koch SE, Rubinstein J, Heiny JA, et al. Dilated cardiomyopathy-mediated heart failure induces a unique skeletal muscle myopathy with inflammation. Skelet Muscle. 2019;9:4. doi:10.1186/s13395-019-0189-y

S2. Brooks SV, Faulkner JA. Contractile properties of skeletal muscles from young, adult and aged mice. J Physiol. 1988;404:71-82. doi:10.1113/jphysiol.1988.sp017279

S3. Zhou Y, Zhou B, Pache L, Chang M, Khodabakhshi AH, Tanaseichuk O, et al. Metascape provides a biologist-oriented resource for the analysis of systems-level datasets. Nat Commun. 2019;10:1523. doi:10.1038/s41467-019-09234-6

S4. Trappe S, Williamson D, Godard M. Maintenance of whole muscle strength and size following resistance training in older men. J Gerontol A Biol Sci Med Sci. 2002;57:B138-43. doi:10.1093/gerona/57.4.b138

S5. Kuster DW, Cardenas-Ospina A, Miller L, Liebetrau C, Troidl C, Nef HM, et al. Release kinetics of circulating cardiac myosin binding protein-C following cardiac injury. Am J Physiol Heart Circ Physiol. 2014;306:H547-56. doi:10.1152/ajpheart.00846.2013

S6. Ciciliot S, Rossi AC, Dyar KA, Blaauw B, Schiaffino S. Muscle type and fiber type specificity in muscle wasting. Int J Biochem Cell Biol. 2013;45:2191-9. doi:10.1016/j.biocel.2013.05.016
